# Mediator Subunit Med15 Regulates Cell Morphology and Mating in *Candida lusitaniae*

**DOI:** 10.3390/jof9030333

**Published:** 2023-03-08

**Authors:** Ayman Sabra, Nicolas Biteau, Jean-William Dupuy, Christophe Klopp, Thierry Noël, Karine Dementhon

**Affiliations:** 1 Vaccines Medical Department, Pfizer, 23-25 Av du Dr Lannelongue, F-75014 Paris, France; 2 Univ. Bordeaux, CNRS, Microbiologie Fondamentale et Pathogénicité, UMR 5234, F-33000 Bordeaux, France; 3 Univ. Bordeaux, Plateforme Protéome, F-33000 Bordeaux, France; 4 Plateforme Bioinformatique Genotoul, BioinfoMics, UR875 Biométrie et Intelligence Artificielle, INRAE, F-31326 Castanet-Tolosan, France

**Keywords:** mediator, *MED15/GAL11*, *Candida lusitaniae*, opportunistic pathogenic yeast, *DPP3*, mating, hyphal growth, cell separation, additional mutation

## Abstract

*Candida lusitaniae* is an emerging opportunistic pathogenic yeast capable of shifting from yeast to pseudohyphae form, and it is one of the few *Candida* species with the ability to reproduce sexually. In this study, we showed that a *dpp3*Δ mutant, inactivated for a putative pyrophosphatase, is impaired in cell separation, pseudohyphal growth and mating. The defective phenotypes were not restored after the reconstruction of a wild-type *DPP3* locus, reinforcing the hypothesis of the presence of an additional mutation that we suspected in our previous study. Genetic crosses and genome sequencing identified an additional mutation in *MED15*, encoding a subunit of the mediator complex that functions as a general transcriptional co-activator in Eukaryotes. We confirmed that inactivation of *MED15* was responsible for the defective phenotypes by rescuing the *dpp3*Δ mutant with a wild-type copy of *MED15* and constructing a *med15*Δ knockout mutant that mimics the phenotypes of *dpp3*Δ in vitro. Proteomic analyses revealed the biological processes under the control of Med15 and involved in hyphal growth, cell separation and mating. This is the first description of the functions of *MED15* in the regulation of hyphal growth, cell separation and mating, and the pathways involved in *C. lusitaniae*.

## 1. Introduction

*Candida* yeasts account for 70 to 90% of all human invasive fungal infections [1]. Candidemia are associated with the highest mortality rates among all bloodstream infections, exceeding 50% in intensive care units despite the use of appropriate treatment [2,3]. As opportunistic pathogens, *Candida* yeasts can take advantage of a deficient immune system or a breach in the skin or mucosal barriers to become invasive. *Candida albicans* is the most common cause of candidiasis; therefore, it is the most studied of all *Candida* species. Nevertheless, over the past two decades, the prevalence of *C. albicans* has gradually decreased in several countries around the world, and non-albicans species are now responsible for half of all reported candidemia episodes [4,5,6,7]. *Candida* (teleomorph *Clavispora*) *lusitaniae* is known as an environmental species, but it can be recovered from the human gastrointestinal tract [8]. Although *C. lusitaniae* is much less common than other *Candida* species (1–3% of all cases of candidemia) [9,10], it has long been reported to cause human infections, particularly in pediatrics and cancer patients [10,11,12]. *Candida lusitaniae* has been classified as an emerging opportunistic pathogen [13] and should not be underestimated due to its ability to develop resistance to multiple antifungals during treatment [10,14,15], which represents a major challenge during clinical management. *Candida lusitaniae* is part of the CTG clade of *Saccharomycotina* (so called because it translates CTG codons as serine instead of leucine) and is phylogenetically related to *C. auris*, a newly emerging pathogen that is highly resistant to antifungals and responsible for nosocomial outbreaks in several countries [16,17]. *C. lusitaniae* is a dimorphic yeast, capable of switching from yeast to pseudohyphal form. It is capable of growing both anaerobically and aerobically, similar to other *Candida* species. *C. lusitaniae* has the advantages of being a haploid yeast that has a sequenced genome [18] and a controllable sexual cycle in vitro [19,20,21,22]. In addition, molecular tools have been developed to make this yeast an attractive model for studying gene function by reverse genetics [23,24,25,26].

In a previous study, we inactivated the *DPP3* gene in *C. lusitaniae* [25]. *DPP3* encodes a farnesyl pyrophosphatase in *C. albicans* [27]. We showed that the *dpp3*Δ knockout mutant in *C. lusitaniae* shared some common phenotypes with its knockout counterpart in *C. albicans* (e.g., reduced intracellular pyrophosphate and a lack of virulence in mice), while it diverged in others (e.g., the deregulation of signaling molecules secretion, as well as its interaction with macrophages). Our findings highlighted that sequence homology between orthologous genes is not synonymous with the conservation of function. Interestingly, the reintroduction of a WT copy of *DPP3* into the *dpp3*Δ knockout mutant was sufficient to restore WT phenotypes in vivo (although not completely) but was not sufficient to restore WT phenotypes in vitro. To circumvent any issue related to a specific molecular technique, an independent *dpp3*Δ knockout mutant was generated, and four different methods were used to reinsert a wild-type copy of the *DPP3* gene [25]. Both *dpp3*Δ knockout mutants exhibited the same phenotypes in vitro and neither reconstituted strain had its phenotypes restored. At this point, we hypothesized that an additional mutation may have occurred in the yeast upon inactivation of the *DPP3* gene, and that this mutation led to the phenotypes observed in vitro.

In this study, we intended to further characterize the *dpp3*Δ mutant with respect to cell physiology. We found that *dpp3*Δ knockout mutants were affected for cell separation, pseudohyphae formation and mating. Again, wild-type phenotypes were not restored when a wild-type copy of *DPP3* was reintroduced into the *dpp3*Δ mutants, reinforcing the hypothesis of an additional mutation. Our main objective was to identify the suspected mutation and characterize the biological processes associated with the phenotypes. To this end, we undertook genetic and proteomic analyses. We found that the defective phenotypes in cell separation, hyphal growth and mating were not linked to the *dpp3*Δ mutation itself but to an additional mutation that occurred in the gene encoding the Med15/Gal11 mediator subunit.

## 2. Materials and Methods

### 2.1. Strains, Media and Growth Conditions

*Candida lusitaniae* CBS 6936 *MAT***a** (ATCC 38533) was used as the wild-type (WT) strain in all experiments. Two independent *dpp3*Δ knockout mutants and four strains reconstituted for a *DPP3* wild-type locus that we previously generated [25] were used for cell morphology and mating phenotyping. Strains 5094 *MAT*α (Centraalbureau voor Schimmelcultures -CBS-, Baarn, The Netherlands) and CL38 *MAT*α (clinical strain) were used as compatible mating partners. The *ura3*Δ *leu2*Δ *MAT***a** strain [24] was crossed with the 5094 *MAT*α strain, and a *leu2*Δ *MAT*α progeny was selected for quantitative mating tests. All strains used in this study are listed in Table 1.

Yeasts were grown in YPD-rich medium (1% yeast extract, 2% bactopeptone, 2% glucose). The minimal-medium YNB (yeast nitrogen base) without amino acid (Difco) supplemented with 2% glucose was used to select prototrophic strains. YCB (yeast carbon base, Difco) medium containing no nitrogen source was used for pseudohyphae growth and mating assays. Selection of yeast transformants after integration of the *URA3* marker was performed on YNB supplemented with 1 M sorbitol. Counter-selection after the *URA3* marker excision in the transformants was performed on YNB without amino acid supplemented with 1 mg/mL 5-fluoroorotic acid (5-FOA; Sigma Chemicals Co., St. Louis, MO, USA) and 50 μg/mL uracil [23,24].

*E. coli* Stellar™ bacteria (kit Clontech) were used for plasmidic DNA transformation. Transformants were selected on LAXI solid medium (LB supplemented with 100 µg/mL ampicillin, 0.5 mM IPTG and 75 µg/mL X-Gal). Recombinant clones were grown in LB liquid medium supplemented with 50 µg/mL ampicillin at 37 °C under 220 rpm.

### 2.2. Pseudohyphal Growth Assays

After overnight culture in YPD, 2 × 10^8^ yeasts were washed and resuspended in 100 µL of sterile water. Aliquots of 5 µL were then spotted onto YCB agar plates 1 cm from the edges of the plates. When needed, plates were supplemented with uracil (25 µg/mL) or farnesol (Sigma) (0.1 µM to 200 µM, stored at a final concentration of 1 mM in ethanol at −20 °C). Stock solutions were brought to room temperature and aliquoted into empty Petri dishes at different concentrations. Liquid medium (50 °C) was added and the plates were swirled to mix. Pseudohyphal growth of each strain was measured daily during one week of incubation at 30 °C.

### 2.3. Mating Assays

Mating assays were performed on YCB agar plates as described previously [20,28]. To test their mating ability, *MAT***a** strains were crossed with a tester strain of the opposite *MAT*α mating type (5094 or CL38) (see Table 1). Quantitative matings were performed by crossing either the *ura3*Δ, *MAT***a** strain [24] or the *ura3*Δ, *dpp3*Δ::*0, MAT***a** strain [25] with the *leu2*Δ *MAT*α strain (this study). After 48 h, mating cells were suspended in 1 mL of 50 mM phosphate buffer (NaHPO_4_, pH 6.5, 1 M Sorbitol). In total, 10 µL of β-mercaptoethanol (Aldrich^®^ Chemistry) and 6 µL of Zymolyase 20T (Seikagaku Biobusiness) (125 mg/mL) were added and the yeast suspensions were incubated for 1 h at 37 °C. After centrifugation (10 min, 3000× *g*), the pellets were resuspended in 1 mL of 0.2% SDS and incubated for 1 h at 37 °C. This treatment kills most of the parental yeast cells but does not damage the more resistant yeast ascospores. The yeasts were then washed twice in water and plated on YNB minimal medium. This ensured that no remaining auxotrophic parental strains could grow and that only ascospores prototrophic for both leucine and uracil were selected and counted. For mating polarity assays, only one of the two partners was stained during overnight culture in YPD with 5 µg/mL Calcofluor White (CFW) (Sigma). The next day, the cells were washed with sterile water before mating assays were performed.

### 2.4. Microscopy

A Zeiss Axioplan microscope with a Micromax camera (Princeton Instruments, Trenton, NJ, USA), a Nikon Eclipse 50*i* microscope with a digital camera, and an EVOS f1 AMG microscope were used to visualize the yeast cells and capture images.

### 2.5. Preparation of Candida Protein Extracts for Proteomic Analysis

Total protein extracts were prepared from three different experiments for each strain. For total lysates, cells were grown in YPD for 16 h at 35 °C and washed in water. The cell pellets were resuspended in 500 μL of lysis buffer (250 mM Tris-HCl pH 6.8, 8% SDS, 40% glycerol, 0.5 mg/mL bromophenol blue, 5% β-mercaptoethanol). An equal volume of 0.4–0.6 mm diameter acid-washed glass beads (SIGMA) was added. Cells were ground in a Cell Breaker (Precellys) for 40 s and cooled on ice for 5 min. This step was repeated, and the lysate was incubated in a water bath at 100 °C for 5 min. A 200 μL volume of proteic extracts was collected and frozen at −20 °C.

### 2.6. Label-Free Quantitative Proteomics

Proteins were loaded onto a 10% acrylamide SDS-PAGE gel. Migration was stopped when the samples entered the resolving gel, and the proteins were visualized by Colloidal Blue staining. Protein digestion and analyses by nano-liquid chromatography–tandem mass spectrometry on Q Exactive were performed as previously described [29]. Protein identification and Label-Free Quantification (LFQ) were performed in Proteome Discoverer 2.3. MS Amanda 2.0, Sequest HT, and Mascot 2.4 algorithms were used for protein identification in batch mode by searching against a NCBI database of *Clavispora lusitaniae* strain CBS6936 (ASM167369v2, 5537 entries, released 8 June 2017). Two missed enzyme cleavages by trypsin were allowed. Mass tolerances in MS and MS/MS were set at 10 ppm and 0.02 Da. Oxidation (M), acetylation (K) and deamidation (N, Q) were sought as dynamic modifications, and carbamidomethylation (C) was sought as static modification. Peptide validation was performed using the Percolator algorithm [30], and only “high confidence” peptides were retained, corresponding to a 1% false discovery rate at the peptide level. Minora feature detector node (LFQ) was used along with the feature mapper and precursor ions quantifier. Normalization parameters were selected as follows: (1) unique peptides; (2) precursor abundance based on intensity; (3) normalization mode: total peptide amount; (4) protein abundance calculation: summed abundances; (5) protein ratio calculation: pairwise-ratio-based; (6) Hypothesis test: *t*-test (background-based). Quantitative data were considered for master proteins, quantified by a minimum of 2 unique peptides, a fold change greater than 2, and a statistical *p*-value lower than 0.05. Statistical *p*-values were adjusted using the Benjamini–Hochberg correction for a FDR lower than 0.05. Mass spectrometry proteomics data were deposited to the ProteomeXchange Consortium (http://proteomecentral.proteomexchange.org, accessed on 6 July 2020) via the PRIDE partner repository [31] with the dataset identifier PXD020220.

### 2.7. Genome Sequencing, Assembly and Identification of Genetic Modifications

DNA libraries of the *ura3*Δ recipient strain and the *dpp3*Δ mutant (*ura3*Δ::*URA3, dpp3*Δ::*0*, *MAT***a**) were prepared from 1 µg according to the NEBNext DNA library prep master mix set for the Illumina (E6040) protocol with an insert size of 368 +/− 122 nucleotides generated with a Covaris ultrasonicator. Libraries were sequenced on the Illumina MiSeq version 1.18.54 platform using the same strategy as for the WT 6936 [18]. The mutant strains raw reads were then aligned against the 6936 genomes, which was used as a reference for searching for genetic modifications (indels, SNP). Each pair of read files was aligned against the wild-type reference with bwa mem [32] (version 0.7.12-r1039) using default parameters. The resulting alignment files were compressed, sorted and indexed with samtools [33] (Version: 1.1) view, sort and index modules, using default parameters. Read groups were added to the alignment files and duplicates marked with the picard-tools modules AddOrReplaceReadGroups.jar and MarkDuplicates.jar (“Picard Toolkit.” 2018. Broad Institute, GitHub Repository. http://broadinstitute.github.io/picard/, accessed on 16 February 2018; version 1.88). The bam files were then merged with samtools and processed with GATK [34] (version 3.7) to realign sequence parts located at indel boundaries and recalibrate nucleotide quality values and call variants. Variants were annotated using snpEFF [35] (version 4.2). The maximum size for indel detection was set to 50 nt. The result file was filtered to extract variants showing specific alleles of *dpp3*Δ compared with the wild-type.

### 2.8. Genotyping and Sequencing of the DPP3 and MED15 Loci

Primer pair 5AmDpp3_800 and 3AvDpp3_521 was used to amplify the *DPP3* locus in the WT or the *dpp3*Δ strain, and amplified a band of about 2.2 kbp or 1.2 kbp, respectively. Primers B584 and B586 were used to amplify and detect the 19 bp insertion in the mutated *MED15* allele. All primer sequences are given in Table S1 in Supplementary Material.

### 2.9. med15 Knockout and Reconstituted Wild-Type MED15 Strains

A *med15*Δ mutant (*ura3*Δ, *med15*Δ::*URA3*, *MAT***a**) was created by integration of an inactivation cassette by double homologous recombination at the *MED15* locus, which replaces the gene and complements the auxotrophy of the *ura3*Δ recipient strain. The inactivation cassette consists of the selective marker *URA3* (encoding oroditine-5-phosphate decarboxylase in the de novo uracil synthetic pathway) flanked by the upstream and downstream regions of *MED15*. The cassette was constructed using the In-Fusion® multiple fragment cloning kit (Clontech). Primers B413 and B414 were used to amplify the *MED15* upstream region. Primers B415 and B416 were used to amplify the *MED15* downstream region. Primers B280 and B238 were used to amplify the *URA3* fragment. The three overlapping PCR fragments were recombined and cloned into the pUC19 plasmid (Promega). Primers B343 and B344 were used to amplify the final *MED15* inactivation cassette that was transformed into the recipient *C. lusitaniae ura3*Δ strain by electroporation. PCR amplification and yeast transformation were conducted according to the manufacturer’s instructions and as previously described [24]. A wild-type fragment of *MED15*, amplified using B419 and B420 primers, was reinserted into the *med15*Δ mutant to obtain the reconstituted strain (*ura3*Δ, *med15*Δ::*MED15*, *MAT***a**). The same strategy was employed to insert the inactivation cassette containing the *URA3* marker in place of the 19 bp insertion in the *dpp3*Δ *ura3*Δ mutant *(ura3*Δ, *dpp3*Δ::0, *med15*^mut^ *MAT***a**), thus creating the *dpp3*Δ *med15*Δ mutant (*ura3*Δ, *dpp3*Δ::0, *med15*Δ::*URA3, MAT***a**) expressing the same truncated Med15 protein. This allowed us, using the 5-FOA counterselection, to reinsert a WT copy of *MED15* at the mutated *MED15* locus of the *dpp3*Δ *med15*Δ mutant, thus creating the reconstituted *dpp3*Δ *med15*Δ + *MED15* strain (*ura3*Δ, *dpp3*Δ::*0*, *med15*Δ::*MED15, MAT***a**). All constructed strains were verified by PCR and sequencing and are listed in Table 1. All the primers used in this study are listed in Table S1 in the Supplementary Material.

### 2.10. Statistical Analysis of the Data

Statistical significance was assessed using analysis of variance (ANOVA https://biostatgv.sentiweb.fr/?module=tests/anova, accessed on 21 February 2023) and *t* tests. Differences were considered significant at *p* values < 0.05.

## 3. Results

### 3.1. The dpp3Δ Mutant Is Impaired in Cell Separation

In a previous study, we generated independent *dpp3*Δ mutants using two different strategies: the *dpp3*Δ^GD^ *ura3*Δ mutant was obtained by gene disruption, while the *dpp3*Δ mutant was obtained by gene replacement (Table 1) [25]. Both mutants were analyzed and had the same phenotypes, so in this study we only show data corresponding to the *dpp3*Δ mutant. The *dpp3*Δ and the wild-type (WT) strains had similar growth rates in all media used for experimentation [25].

**Figure 1:**
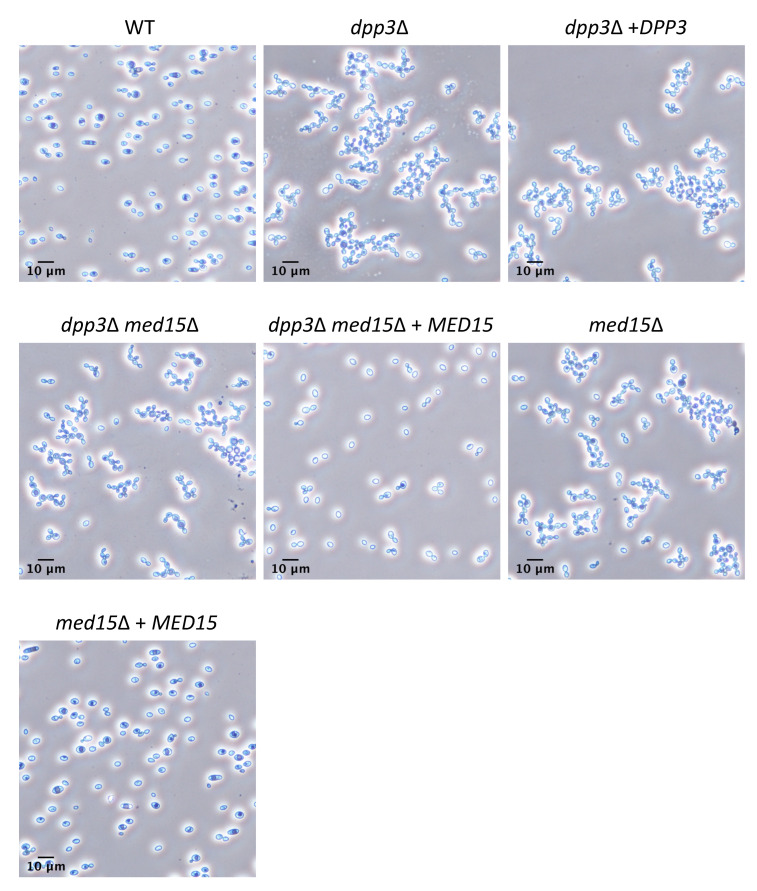
The cell separation defect in the *dpp3*Δ mutant is due to *med15*Δ mutation. WT and mutant strains of *C. lusitaniae* were grown in liquid YPD medium at 35 °C under 215 rpm. Cells were visualized and imaged after 48 h using 40× magnification under brightfield. Scale bars represent 10 µm. Please refer to Table 1 for detailed genotypes of the strains. When a WT copy a *MED15* was reintegrated into the *dpp3*Δ mutant, cell separation returned to wild-type levels.

To compare the morphology of the WT and the *dpp3*Δ mutant, we inoculated 10^6^ cells/mL in liquid YPD. We found that unlike the WT strain, which was mostly under single- or two-cell forms, the *dpp3*Δ mutant formed multi-cell chains, a phenotype indicative of a defect in cell separation (Figure 1).

Observations at earlier time points showed that yeast cells started to multiply by budding and formed small chains of cells for both WT and *dpp3*Δ strains (T 3 h). The cell separation defect of the *dpp3*Δ mutant was also observed at T 6 h and T 24 h (Figure S1A). This cell separation defect leads to cells that remain attached to the mother cells, as evidenced by staining with Calcofluor White (Figure S1B), whose fluorescence is most intense at the cellular bud necks and at the separation between the mother and the daughter cell. This suggests that the clusters are more likely to result from the non-dissociation of mother and daughter cells following cell division rather than resulting from the adherence of random cells to each other.

### 3.2. The dpp3Δ Mutant Exhibits Impaired Pseudohyphal Growth

**Figure 2:**
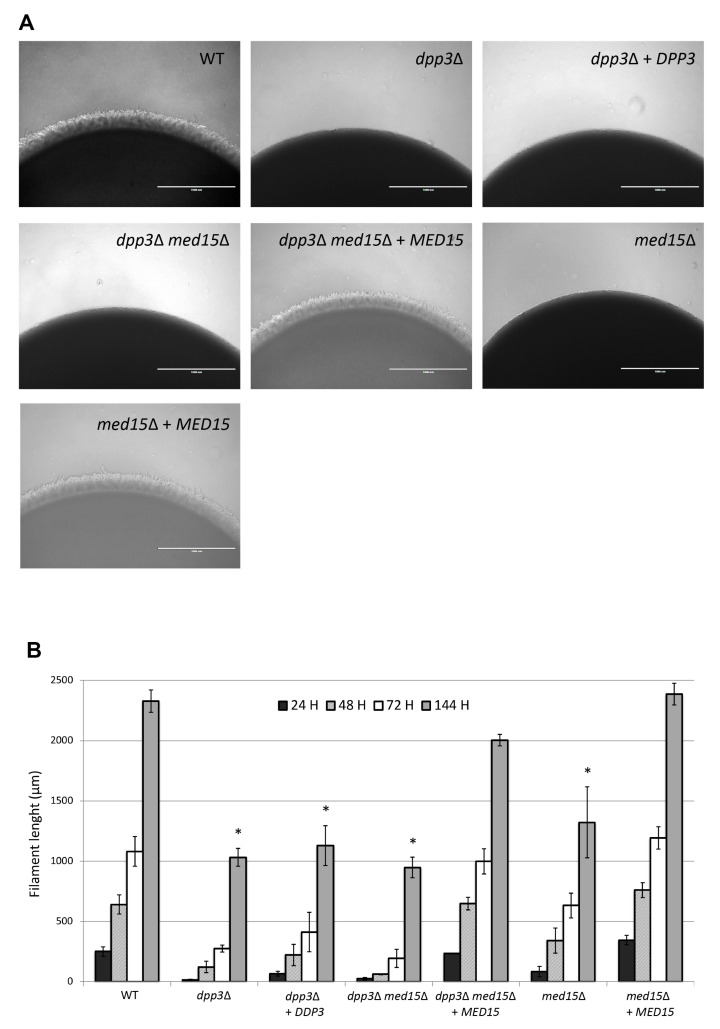
The pseudohyphal growth defect in the *dpp3*Δ mutant is conferred by the *med15*Δ mutation. (**A**) Strains were grown on YCB plates supplemented with uracil at 30 °C. Colony periphery was visualized at 4× magnification under brightfield and photographed after 24 h. Scale bars represent 1 mm. (**B**) Filament length was measured at 24 h, 48 h, 72 h and 144 h using Image J. The average length for one experiment was obtained from 20 measurements for each strain. Results are expressed as mean values ± standard error of data from three independent experiments. Values that are statistically different are marked with an asterisk (*p*-value < 0.05). Please refer to Table 1 for detailed strain genotypes. When a WT copy a *MED15* was reintegrated into the *dpp3*Δ mutant, pseudohyphal growth returned to wild-type levels.

*C. lusitaniae* is able to form pseudohyphae when grown on a solid medium depleted of a nitrogen source [36]. In order to compare the pseudohyphal growth of the WT and the *dpp3*Δ strains, we measured the length of the pseudohyphae daily for 6 days at the periphery of colonies growing on YCB agar plates, and a pseudohyphal growth speed was recorded for each strain. We found that the *dpp3*Δ mutant was severely affected in its ability to form pseudohyphae compared with the WT strain, especially during the first 24 h (Figure 2).

The *dpp3*Δ mutant grew at 0.5 μm/h, whereas the WT grew at approximately 10 μm/h. When the colony border cells were observed using a microscope, the very rare structures formed by the *dpp3*Δ mutant were found to be predominantly yeast-like cells (Figure S2). From day 2 to day 6, the *dpp3*Δ mutant started and continued to make pseudohyphae but never caught up with the WT strain. Overall, the growth rate of pseudohyphae was approximately 7 μm/h for the *dpp3*Δ mutant and 16 μm/h for the WT strain. Furthermore, when whole colonies were picked from the surface of the agar plates, we observed that unlike the WT strain, the *dpp3*Δ mutant did not leave traces of embedded pseudohyphae, showing that the few pseudohyphae of the mutant were unable to break through the agar. In conclusion, our data show that the *dpp3*Δ mutant is defective for pseudohyphal growth. Furthermore, as farnesol was shown to inhibit the filamentation process in *C. albicans* on various media commonly used to trigger filamentation [37,38], we tested the effect of adding farnesol on the pseudohyphal growth of *C. lusitaniae* (Figure S3). In contrast to *C. albicans*, the pseudohyphae formation of *C. lusitaniae* was not affected by the presence of farnesol (200 μM) in solid YCB medium (compare panels b and h). Next, we tested the effect of farnesol on the pseudohyphal growth of *C. lusitaniae* in solid RPMI, a medium that alone, does not induce its filamentation. We found that 200 μM of farnesol induced the pseudohyphal growth of the *C. lusitaniae* WT strain (see panel d). In contrast, the *dpp3*Δ mutant did not respond to farnesol (see panel f). These data show that *dpp3*Δ lost its ability to produce pseudohyphae in response to farnesol on solid RPMI.

### 3.3. The dpp3Δ Mutant Has a Defect in Mating

**Figure 3:**
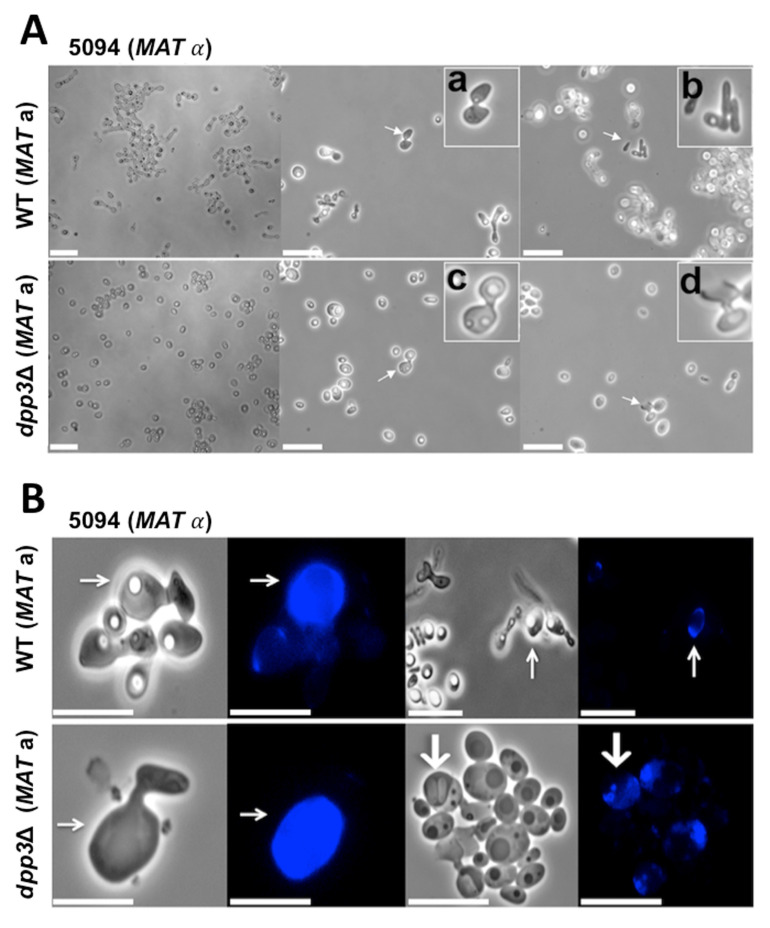
Mating of the WT and *dpp3*Δ strains with the same tester strain. Images were taken after 48 h of incubation at 30 °C. Scale bars represent 10 µm. (**A**) Mating between the 5094 *MAT*α and the WT or *dpp3*Δ *MAT***a** strains. Inserts show enlarged views of the cells indicated by arrows. (**a**,**c**) Sexual conjugation between two yeast cells. (**b**) Ascospores. (**d**) Ascus. (**B**) Mating between the non-stained 5094 *MAT*α and the CFW-stained WT or *dpp3*Δ *MAT***a** strains. Acceptor cells are indicated by thin arrows, and an ascus is indicated by a thick arrow.

**Figure 4:**
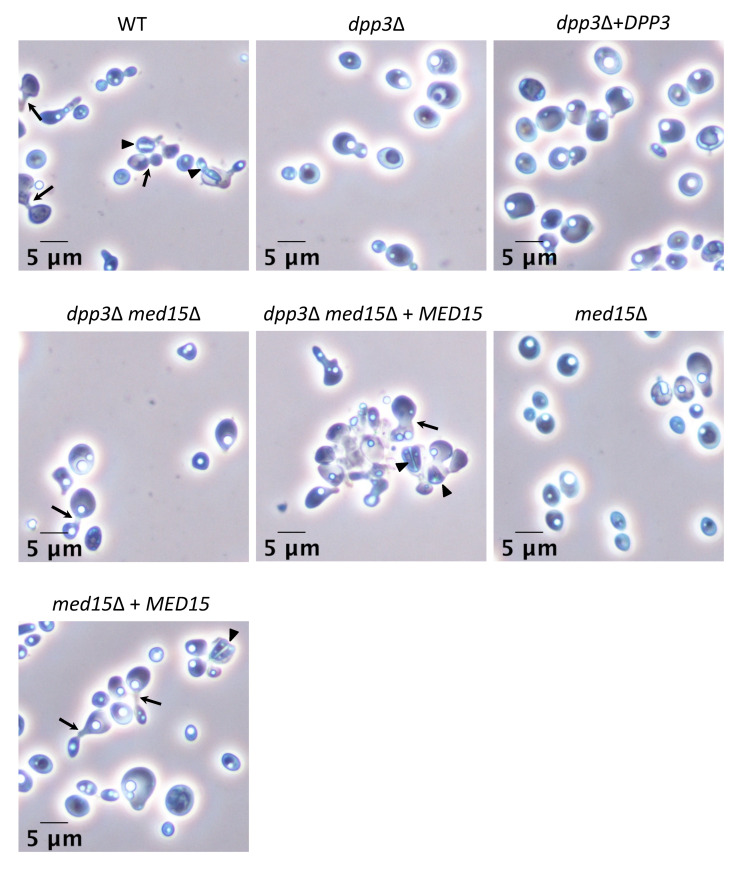
The mating defect in the *dpp3*Δ mutant is conferred by the *med15*Δ mutation. WT and mutant strains of *C. lusitaniae* (*MAT***a**) were crossed with the CL38 strain (*MAT*α). Images were taken after 48 h of incubation at 30 °C. Scale bars represent 5 µm. Asci are indicated by arrowheads, and conjugation tubes by arrows.

Because the mating process in *C. lusitaniae* requires yeasts to grow germ tubes, we tested whether the *dpp3*Δ mutant was affected with respect to its mating ability. The WT strain (*MAT***a**) or the *dpp3*Δ strain (*MAT***a**) was crossed with the same opposite mating-type tester strain 5094 (*MAT*α). When aliquots of mating yeasts were placed on glass slides and observed with the microscope, typical mating structures such as conjugation tubes and ascospores [20] were detected for both strains (Figure 3A). However, the number of yeasts engaged in mating was much lower for the *dpp3*Δ mutant compared with the WT strain. The cross involving the *dpp3*Δ mutant yielded only 5% of the spores produced by the control mating. The defect in the mating of the *dpp3*Δ mutant compared with the WT strain was confirmed in a cross using another *MAT*α tester strain (CL38) (Figure 4). We concluded that the *dpp3*Δ mutant has a “quantitative defect” in sexual reproduction.

During the mating process, nuclear transfer is polarized: one strain acts as a nucleus donor and the other as a nucleus acceptor. The acceptor strain is usually easy to identify as it outgrows the donor strain, turning into an ascus. To test the effect of the mutation on mating polarity, one of the mating partners was stained with CFW prior to experimentation. We found that the polarity of the *dpp3*Δ mutant mating was not affected compared with the WT strain (Figure 3B). Both strains were stained with CFW and acted as acceptor strains when crossed with the unstained, sexually compatible 5094 *MAT*α strain. To ensure that mating polarity was not biased by CFW staining, another crossing experiment was performed, where strain 5094 *MAT*α was stained and crossed with the unstained WT or *dpp3*Δ strains. We found that the mating polarity remained unchanged, e.g., strain 5094 *MAT*α acted as a nuclear donor, and both WT and *dpp3*Δ strains acted as acceptor strains.

### 3.4. Inactivation of the DPP3 Gene Is Not Directly Responsible for Cell Morphology and Mating Defects

In our previous study, four different techniques were used to reinsert a WT copy of the *DPP3* gene into the different knockout mutants. *DPP3* mRNA and protein were detected in the reconstituted (REC) *dpp3*Δ + *DPP3* strains [25]. When we analyzed the REC strains, strikingly, neither cell separation (Figure 1), pseudohyphal growth (Figure 2) nor mating phenotypes (Figure 4) were restored to their WT levels in any of the reconstituted (REC) strains obtained, reinforcing our hypothesis of the presence of an additional mutation in the *dpp3*Δ mutant. We set out to confirm this hypothesis and to identify the suspected mutation. To do so, we first took advantage of the mating ability of *C. lusitaniae* and performed genetic analyses. To determine whether inactivation of the *DPP3* gene conferred the defective phenotypes, we co-analyzed the segregation of the *dpp3*Δ mutation with the segregation of pseudohyphal growth, cell separation and mating phenotypes (Table 2). The *dpp3*Δ *ura3*Δ *MAT***a** mutant was crossed with a *leu2*Δ *MAT*α strain. Prototrophic ascospores for both uracil and leucine were selected on YNB medium to ensure that recombinant progeny were used for genetic analyses. Thus, only one-fourth of the progeny was analyzed (independent chromosomes hold *URA3* and *LEU2* genes). Because the parent mutated for *DPP3* is deficient for mating, despite the fact that the experiment was repeated several times, a total of only 27 ascospores could be recovered. The progenies were typed by PCR at the *DPP3* locus to distinguish the wild-type from the mutated allele, and they were analyzed for each phenotype of interest. We reasoned that if *DPP3* inactivation was directly responsible for the defective phenotype, this phenotype should always co-segregate with *dpp3*Δ mutation. Strikingly, we found descendants carrying the mutated *DPP3* gene but exhibiting WT cell separation, pseudohyphae formation and mating, and vice versa, there were descendants carrying a WT copy of *DPP3* that were defective in each of the phenotypes (Table 2). These data show that inactivation of the *DPP3* gene is not directly responsible for the defective phenotypes, and that the *dpp3*Δ knockout carries another mutation elsewhere in the genome, which is responsible for these phenotypes. Furthermore, we found that the mating, pseudohyphal growth and cell separation defects always co-segregated in the descendants, suggesting that the gene affected by the additional mutation regulates these three processes. Finally, although the majority (22/27, or ≈80%) of the progeny was haploïd, some (5/27, or ≈20%) carried both parental alleles at the *DPP3* locus, suggesting aneuploïdy or diploïdy, as previously described [21]. In an attempt to identify the additional mutation to *DPP3* inactivation, we sequenced the genome of the *dpp3*Δ mutant.

### 3.5. The dpp3Δ Mutant Carries an Additional Mutation in the MED15 Gene

To assess the presence of additional mutations in the *dpp3*Δ mutant, we compared the genome of the *dpp3*Δ mutant with the genomes of the *ura3*Δ recipient strain and the WT strain (same genetic backgrounds). The comparison of the genomes of the *ura3*Δ recipient strain and the WT strain confirmed the deletion in the *URA3* ORF, and we detected six variants (single nucleotide polymorphism (SNP), and insertion/deletion or indel), none of which we predicted to have a high impact on the corresponding protein (Table S2.1). Genome analysis of the *dpp3*Δ mutant confirmed on one hand the expected deletion in the *DPP3* gene, as well as the reconstituted wild-type *URA3* locus. On the other hand, we identified six polymorphisms (indels) in the *dpp3*Δ mutant that were not present in the *ura3*Δ recipient strain (Table S2.2). Only one variant was located in an ORF (OVF 06598.1, putatively encoding the Med15/Gal11 subunit of Mediator) with a predicted high impact on the protein composition. This variant is an insertion of a 19 nt sequence (GAGGAATGAACAACATGAG) at CDS position 1076, resulting in a frameshift and a premature stop codon. We confirmed the presence of this predicted mutation in the *dpp3*Δ and the *dpp3*Δ *ura3*Δ mutants, as well as its absence in the WT and the *ura3*Δ recipient strains, by directly sequencing the *MED15* locus. The nucleotide sequence alignment of the mutant and wild-type *MED15* loci is shown in Figure S4A. The alignment of the corresponding proteins showed that the mutated Med15 protein is truncated at amino acid 375 and lacks both the Med15 and PHA03378 domains (Figure S4B). We hypothesized that this mutation would likely affect the function of the Med15 protein and could be responsible for the phenotypes of the *dpp3*Δ that did not return to WT after the reinsertion of a WT copy of *DPP3*. We then sequenced the MED15 locus in the reconstituted *dpp3*Δ + *DPP3* strain. As expected, we found the 19 nt sequence insertion, which was identical to that of the *dpp3*Δ mutant. Similarly, this insertion was found in the *dpp3*Δ *ura3*Δ strain, from which the *dpp3*Δ mutant is derived. Since the other *dpp3*Δ^GD^ *ura3*Δ mutant, obtained in an independent experiment, also did not revert to WT phenotypes after the reconstruction of an intact *DPP3* locus, we wanted to know if the same mutation in *MED15* was present. Again, we found a mutation in the *MED15* gene, but this time it was a 350 nucleotide insertion. We reassigned genotypes to specify the *med15*Δ mutations, as indicated in Table 1. From these data, we concluded that the inactivation of the *DPP3* gene repeatedly resulted, in independent experiments, in the selection of an additional mutation, thereby inactivating *MED15*.

### 3.6. The Mutation in MED15 Is Responsible for Cell Morphology and Mating Defects

To determine whether the *med15*Δ mutation was responsible for the defective phenotypes in the *dpp3*Δ mutant, we first attempted to rescue the *dpp3*Δ *ura3*Δ mutant (*ura3*Δ, *dpp3*Δ::0, *med15*^mut^, *MAT***a**) by integrating a WT copy of the *MED15* gene, thus creating the reconstituted *dpp3*Δ *med15*Δ + *MED15* strain (*ura3*Δ, *dpp3*Δ::*0*, *med15*Δ::*MED15*, *MAT***a**). When we analyzed cell morphology, we found that cell separation (Figure 1) and pseudohyphal growth (Figure 2) returned to wild-type phenotypes. Furthermore, we found that the reconstitution of a WT *MED15* locus in the *dpp3*Δ mutant restored WT mating ability (Figure 4). Next, we inactivated *MED15* in the *ura3*Δ recipient strain to assess the phenotypic consequences of *MED15* inactivation in the absence of the *dpp3*Δ mutation. The *med15*Δ *(ura3*Δ, *med15*Δ::*URA3*, *MAT***a**) mutant phenocopied the *dpp3*Δ mutant and exhibited defective cell separation (Figure 1), pseudohyphae formation (Figure 2) and mating phenotypes (Figure 4). Finally, we reintegrated a WT copy of *MED15* into the *med15*Δ mutant, obtaining the reconstituted *med15*Δ + *MED15* strain (*ura3*Δ, *med15*Δ::*MED15*, *MAT***a**). This reconstituted strain reverted to WT phenotypes (Figure 1, Figure 2 and Figure 4). All together, our data confirm that the previously unknown mutation in *MED15* that occurred in the *dpp3*Δ strain is the one responsible for the defective cell morphology and mating phenotypes of the mutant. A summary of all phenotypes of the *dpp3*Δ mutant examined in this work and in our previous study, as well the contributions of the *dpp3*Δ and *med15*Δ mutations, is provided in Table S5.

### 3.7. Protein Profile Changes Due to dpp3Δ and med15Δ Mutations

To characterize the proteins and pathways associated with the defective phenotypes related to the *dpp3*Δ and/or *med15*Δ mutations, we performed a label-free proteomic analysis of the *dpp3*Δ mutant (*ura3*Δ::*URA3, dpp3*Δ::0, *med15*^mut^ *MAT***a***)*, the reconstituted strain (*ura3*Δ::*URA3, dpp3*::*DPP3, med15*^mut^, *MAT***a**) and the WT strain (6936). Given the discovered *med15*Δ mutation, the changes in the protein profiles in the *dpp3*Δ mutant are therefore attributable to the mutation in the *DPP3* gene and/or the mutation in the *MED15* genes, and in the REC strain to the *med15*Δ mutation alone. Three independent replicates of experiments for each strain were performed, and 2640 proteins were detected (with a minimum of two unique and specific peptides), representing approximately 48% of the cellular proteome. Only proteins showing differential expression with a statistically significant ratio greater than or equal to two were analyzed.

**Figure 5:**
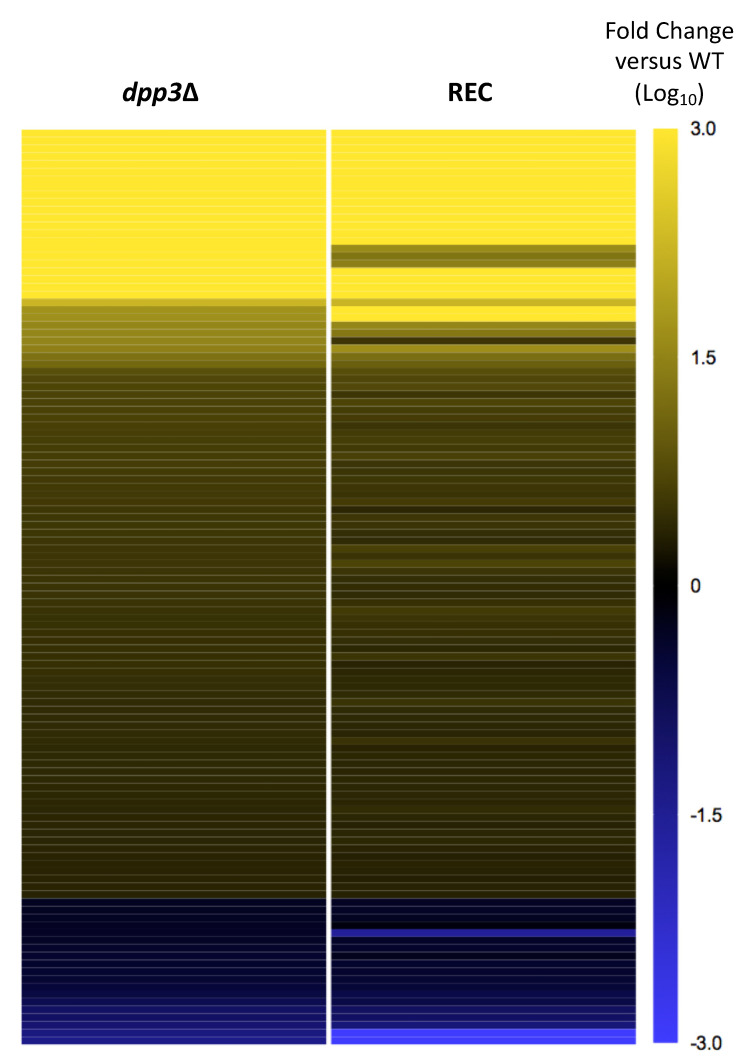
Heat map showing the REC strain the expression profile of the proteins deregulated at least 2-fold (*p* value < 0.05) in the *dpp3*Δ relative to the WT. Proteins that were not quantified in at least two biological replicates out of the three in both the *dpp3*Δ and the REC strains were taken out of the analysis. The heat map was generated in Prism8. The fold change (Log_10_) relative to the WT is color coded as indicated in the side panel. The proteins deregulated in the *dpp3*Δ mutant were still deregulated in the REC strain despite a wild-type copy of *DPP3* being reintegrated, likely reflecting changes attributable to *med15*Δ mutation.

A first comparison of the proteome of the *dpp3*Δ mutant with that of the REC strain revealed their strong similarity. Only 45 proteins, of which 24 were up-regulated and 21 were down-regulated, were found to be differentially expressed after the reconstitution of a *DPP3* WT locus, indicating that most of the proteins deregulated in the *dpp3*Δ mutant remained deregulated in the REC strain, and they are most likely attributable to the *med15*Δ mutation (Table S3). Indeed, when we selected the proteins that were deregulated in the *dpp3*Δ mutant to analyze their expression ratio in the REC strain compared with the WT strain (with a *p*-value < 0.05), none of the 119 proteins returned to their WT level in the REC strain reconstituted for a wild-type *DPP3* locus (Figure 5).

**Figure 6:**
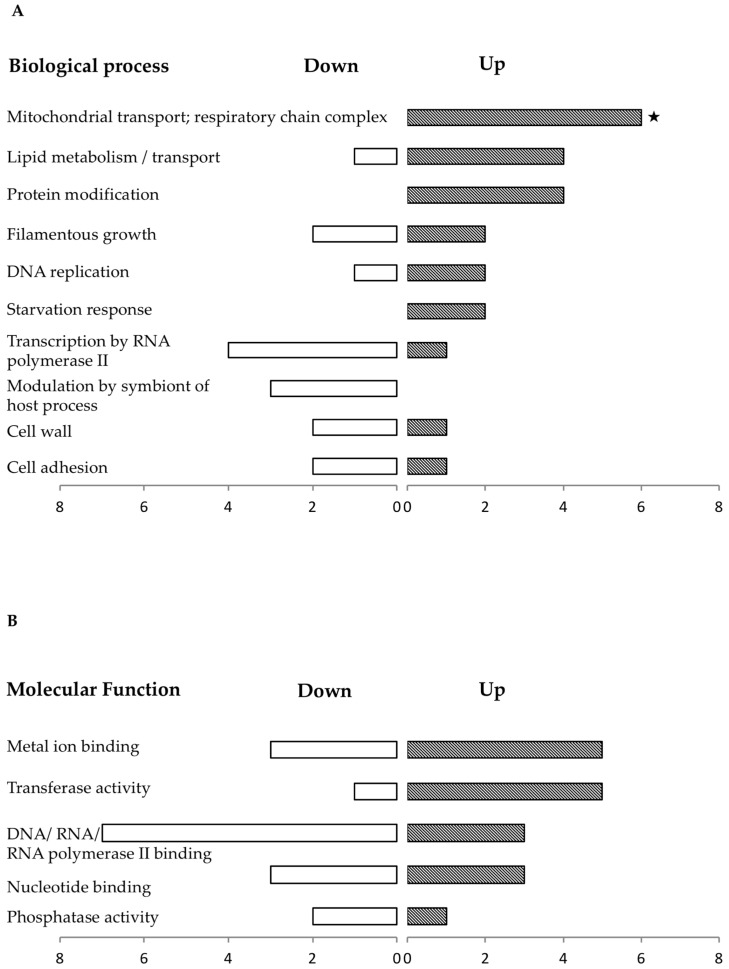
Functional distribution of the proteins found differentially expressed in the dpp3delta mutant compared with the REC strain. Proteins were classified according to the biological processes (**A**) or molecular functions (**B**) with which they are associated based on their predicted functions using gene ontology (GO) annotations of *C. lusitaniae* (ATCC42720) and *C. albicans* (SC5314) in the Uniprot database. The number of proteins down-regulated or up-regulated can be read on the horizontal axis. Since proteins can be linked to more than one annotation group, the sum of annotated genes is larger than the number of total up- and down-regulated genes in the set analyzed. The categories found statistically enriched by the DAVID tool (https://david.ncifcrf.gov, accessed on 28 October 2022) are marked with an asterisk (*p*-value < 0.05). Because the analysis was performed using *C. lusitaniae* reference genome ATCC42720, enrichment of some biological processes annotated from *C. albicans* SC5314 could not be addressed.

The 45 proteins whose expression changed in the REC strain are likely to be proteins under the control of Dpp3, which would not be involved in the still-defective phenotypes in the REC strain (Table S3). An analysis of the 24 up-regulated proteins revealed changes in proteins associated with mitochondrial transport and respiratory chains (e.g., the hypothetical protein OVF05460.1; orf19.4825 involved in the ATP synthase complex assembly, and Tim9 and Tim10 mitochondrial inner-membrane translocase subunits), protein modification (e.g., the ubiquitin-conjugating enzyme Ubc4) and lipid metabolism (e.g., the phosphatidate cytidylyltransferase Pth1). Proteins related to starvation response were also found up-regulated, notably the fructose 1,6-biphosphatase Fbp1 (Figure 6). The down-regulated proteins were mostly related to transcription by RNA polymerase II (e.g., the RNA polymerase subunit orf19.2687.1 and the transcription factor IID subunit OVF07301.1; orf19.1574) and modulation by the symbiont of the host process (e.g., transcription factor Cap1). Proteins involved in filamentous growth were found among the up-regulated proteins (e.g., the calmodulin Cmd1 and the increased rDNA-silencing protein Irs4), as well as among the down-regulated proteins (e.g., the GTPase Arf2 and the mitochondrial protein Mcu1) (Figure 6). DAVID analysis revealed that the only biological process found enriched in the set of proteins under the control of Dpp3 was mitochondrial importation into mitochondria (about 57-fold with a *p*-value = 3.2 × 10^−3^).

**Figure 7:**
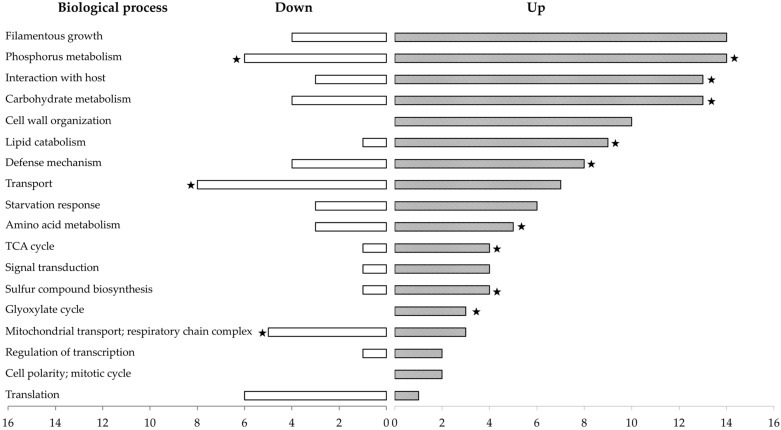
Functional distribution of the proteins found differentially expressed in the REC strain (carrying the *med15*Δ mutation) compared with the WT (6936) strain. Proteins were classified in biological processes based on their predicted functions using gene ontology (GO) annotations from *C. lusitaniae* (ATCC42720) and *C. albicans* (SC5314) in Uniprot database. The number of proteins down-regulated or up-regulated can be read on the horizontal axis. Since proteins can be linked to more than one annotation group, the sum of annotated genes is larger than the number of total up- and down-regulated genes in the set analyzed. The categories found statistically enriched by DAVID tool (https://david.ncifcrf.gov, accessed on 21 February 2023) are marked with an asterisk (*p*-value < 0.05). Because the analysis was performed using *C. lusitaniae* reference genome ATCC42720, enrichment of some biological processes annotated from *C. albicans* SC5314 could not be addressed.

Next, the proteome of the REC strain was further analyzed in comparison with that of the WT strain to uncover the deregulated metabolic pathways associated with the defective phenotype in vitro, which was attributable to the *med15*Δ mutation. In total, 151 proteins (2.7% of the proteome), of which 118 were up-regulated and 33 down-regulated, showed significant expression changes with ratios ranging from 2 to infinity (Table S4). Differentially expressed proteins were classified into biological process groups based on their predicted functions using the gene ontology annotation resource (http://www.ebi.ac.uk/GOA, accessed on 6 August 2020) in the Uniprot database [39] (Figure 7). Most of the proteins up-regulated were involved in metabolic processes (44%), including (i) phosphorus metabolism, such as the CDK inhibitor Pho81 and the MAP kinase Mkc1; (ii) carbohydrate metabolism, such as the glucokinase Glk1 and the glycerol-3-phosphate dehydrogenase Gpd1; (iii) lipid metabolism, such as the bifunctional hydroxyacyl-CoA dehydrogenase/enoyl-CoA hydratase Fox2, and the acetyl-CoA C-acyltransferase Pot1 involved in fatty acid oxidation, namely the lysophospholipase Plb3; (iv) amino acid metabolism, such as proteins involved in arginine (Arg5,6) and methionine/cysteine biosynthesis (Met3, 10, 14, 15); (v) the tricarboxylic acid cycle, such as the malate dehydrogenases Mdh1 and Mdh2, and the isocitrate dehydrogenase Idp2; (vi) sulfur metabolism (such as enzymes involved in trans-sulfuration or sulfur amino acid biosynthesis; and (vii) the glyoxylate cycle, such as the malate synthase Mls1 (Figure 7). Among the up-regulated proteins, we also found proteins with a role in host interactions, including the secreted aspartyl protease Sap9; proteins associated with filamentous growth (such as the Rim101 and Tcc1 transcription factors); the cell wall (such as Hyr3 and Ywp1); or defense mechanisms (such as the catalase Cat1 and the kinases Cmk1,2 and Mkc1). Other biological processes up-regulated in the context of the *med15*Δ mutation include starvation response (e.g., the transporter Mlt1), signal transduction (e.g., the GTPase-activator protein Rgd1), regulation of transcription by RNA-polymerase II (e.g., Tcc1 transcription factor) and cell polarity (e.g., the kinase Mkc1). On the other hand, we found that 24% of the up-regulated proteins had an unknown function. The 33 down-regulated proteins associated with the *med15*Δ mutation were mainly involved in transport (e.g., the ion permeases Ftr1 and Zrt2), in phosphorus metabolism (e.g., the phosphate transporter Pho84) and in mitochondrial transport or the respiratory chain (e.g., the cytochrome c oxidase Cox13; the translocase subunits Tim9 and Tim10) (Figure 7). Proteins associated with translation were also found down-regulated, such as ribosomal proteins Rps30 and Rps14B.

To further explore the biological meaning of our data set, we performed an enrichment analysis using the DAVID tool [40]. *C. lusitaniae* ATCC42720 was used as a reference genome to identify the biological processes and cellular pathways enriched in our protein lists. Among the proteins up-regulated in the context of the *med15*Δ mutation, we detected an enrichment of proteins associated with defense mechanisms (such as hydrogen peroxide catabolism (about 32-fold) and response to oxidative stress (about 21-fold)), and metabolism, especially with the glyoxylate cycle (about 32-fold), TCA cycle and lipid catabolism (Table 3). These processes are important for the utilization of alternative carbon sources, and are also induced to cope with nutrient starvation. Proteins involved in phosphate-, sulfur-amino-acid- and carbohydrate-related metabolism (especially pentose and glucoronate interconversion), as well as in aerobic respiration, were also found enriched. The set of down-regulated proteins was enriched in proteins related to the mitochondrial inner membrane (about 93-fold), ion transport, or metabolism linked to nucleoside, purine or ribose phosphate. Our data suggest that these biological processes under the control of Med15 are associated with the defects in cell separation, hyphal growth and mating of the *dpp3*Δ mutant in *C. lusitaniae*.

## 4. Discussion

In a previous study, we investigated the role of the *DPP3* gene in encoding a putative pyrophosphatase in *C. lusitaniae* by gene inactivation. A caveat in our study was that some phenotypes of the *dpp3*Δ mutant were not reverted to their WT levels after reconstitution of a WT *DPP3* locus, suggesting the existence of an additional mutation. In this study, we sought to better characterize the phenotypes of the *dpp3*Δ mutant in terms of cell physiology and to identify the suspected additional mutation. We discovered that the *dpp3*Δ mutant harbored an additional mutation in the *MED15* gene, encoding a subunit of the mediator complex. We showed that the *dpp3*Δ mutant defects in hyphal growth, cell separation and mating are not due to *dpp3*Δ mutation, but rather to *med15*Δ mutation. Indeed, a wild-type copy of *MED15* inserted in the *dpp3*Δ mutant rescued the defective phenotypes to the wild-type levels. Furthermore, inactivating the *MED15* gene in *C. lusitaniae* mimicked the phenotypes we observed for the *dpp3*Δ mutant in vitro. To our knowledge, this is the first description of *MED15* functions in regulating hyphal growth, cell separation and mating in *C. lusitaniae.*

### 4.1. The Mediator of C. lusitaniae Shares Common Fonctions with Other Yeasts

The mediator is a central regulator of RNA-polymerase-II-dependent transcription in Eukaryotes by bridging DNA-bound transcription factors with the core transcription machinery [41]. Mediator functions have been described in animals, yeast and plants. They affect a wide range of cellular processes, including development, the cell cycle, metabolism and stress responses [42,43]. In fungi, the mediator comprises 25 subunits, which are divided into 4 distinct modules termed head, middle, tail and CDK 8 kinase. The four-module organization and the overall subunit composition of the mediator complex is remarkably conserved throughout evolution, from protists to humans [44]. Med15 is part of the mediator tail. Our data highlighted the functions of *MED15* in regulating *C. lusitaniae* hyphal growth, cell separation and mating. Such pleiotropic effects have been described in other yeast. For example, *S. cerevisiae med15* mutants have impaired growth on alternative carbon sources, such as galactose and fatty acid, as well as impaired mating [45,46]. The *C. lusitaniae* mediator also shares some common functions with that of *C. albicans* and *C. dubliniensis* (reviewed in [47]). In *C. albicans*, the *med15*Δ/Δ mutant is impaired in white–opaque switching [48], a phenotypic transition involved in mating [49]. A mutant of another subunit of the mediator tail, *med3*Δ/Δ, is also impaired for filamentation and mating [48], and is avirulent in mice [50]. In *C. glabrata*, Med15 has been shown to have a role in multidrug resistance [51]. In *C. lusitaniae*, the role of Med15 in drug resistance remains to be further studied. In our previous study, the *dpp3*Δ mutant was reconstructed for a WT copy of *DPP3* carrying the mutation in *MED15*, and it was restored to the WT levels of virulence in mice to a large extent (about 70%) but not to 100%. This lack of full restoration could be due to the *med15*Δ mutation, suggesting that Med15 may contribute to, but not be essential for, wild-type virulence. The actual contribution of Med15 to virulence remains to be demonstrated. In *C. dubliniensis*, the TLO genes encode orthologs of *MED2*, a tail subunit. The *tlo1*Δ/*tlo2*Δ mutants are unable to filament and are defective for cell separation [52]. Our data suggest a conservation of at least some functions of the mediator among yeast.

Mediator positively or negatively controls gene expression, and different subunits regulate specific downstream targets. An analysis of *med15* mutants in *S. cerevisiae* revealed that the Med15 subunit has both positive and negative effects on gene expression. In *C. lusitaniae*, we found that most of the genes (78%) were up-regulated in the strain carrying the mutation in *MED15*, indicating a mainly negative regulation of downstream targets. The relative small fraction of the genome affected by the *med15*Δ mutation in *C. lusitaniae* (about 2.8% showed at least a 2-fold change in protein level) is comparable to what was described in *S. cerevisiae*, where 5–10% of the yeast genome showed at least a 1.5-fold altered transcript level [53]. These findings are consistent with the idea that the tail module is not a general regulator of transcription but rather a regulator of specific genes [54]. Furthermore, Med15 was shown to be mainly dedicated to the activation of highly regulated TATA-containing, SAGA-dependent genes, including genes responsive to environmental and metabolic stress [55]. Enrichment analysis indicated that proteins with expressions significantly affected in the *med15* mutant were mainly related to metabolism and stress response. Glyoxylate cycle, TCA cycle, lipid and amino acid catabolism were found up-regulated. These biological processes allow yeast to use alternative carbon sources and to cope with nutrient starvation. Proteins associated with oxidative stress response were also up-regulated. These results indicate that in *C. lusitaniae* Med15 is involved in nutrient or stress response, in line with the functional conservation of this subunit described in yeasts, worms and humans [42]. For example, Med15 was shown to regulate the expression of genes involved in fatty-acid metabolism by directly interacting through its KIX domain with activators Oaf1, Sbp1 and Srebpα in *S. cerevisiae*, *C. elegans* and humans, respectively [45,56,57]. The KIX domain of Med15 in *S. cerevisiae* was also shown to interact with the Gcn4 activator to induce the expression of amino acid biosynthetic genes [58]. In *C. glabrata*, Med15B inactivation resulted in the deregulation of pathways involved in lipid metabolism under acidic conditions [59]. In *C. dubliniensis*, deletion of another subunit of the tail module in the *tlo1*Δ/*tlo2*Δ mutant resulted in defective filamentous growth and cell separation, similar to what we observed in the *med15*Δ mutant in *C. lusitaniae*. Additionally, as we found in *C. lusitaniae*, the *tlo1*Δ/*tlo2*Δ mutant up-regulated genes involved in the catabolism of alternative carbon and nitrogen sources, such as amino acids and fatty acids [52], confirming a role of the tail module in responding to environmental nutrient status. In the *C. lusitaniae* mutant, the mutation was located downstream the KIX and the Ga111 coactivator domains of the *MED15* gene. While we can not rule out that the truncated protein might be partially functional, we observed phenotypes and modifications of the proteome similar to what is described for other loss-of-function tail-subunit mutants. This suggests that the functions of Med15 in regulating metabolism and stress pathways rely on the Med15 and PHA03378 domains. The down-regulation of import-related proteins in the mitochondrial inner membrane, such as the translocases Tim9 and Tim10, as well as the cytochrome c oxidase Cox13, suggests mitochondrial dysfunction in the *med15*Δ mutant, which is consistent with pleiotropic phenotypes. Interestingly, several proteins whose expression was deregulated in the *med15*Δ mutant were already reported to regulate a function that was found defective in the mutant. For example, the Rho-GTPase-activating protein (RhoGAP) Rgd1, the CDK inhibitor Pho81 and the nitric oxide dioxygenase Yhb1 were found up-regulated in the *med15*Δ mutant in *C. lusitaniae* and were all reported as negative regulators of filamentation in *C. albicans* [60,61,62]. On the other hand, several kinases involved in oxidative stress response and cell wall organization, such as Cmk1 and Cmk2, or cell wall integrity, such as Mkc1, were up-regulated [63,64]. The activation of the cell wall integrity (CWI) pathway via the MAP kinase Mkc1 in response to cell wall stress has been described in *C. albicans*. Furthermore, all cell wall stresses resulted in decreased cell separation and cell wall strengthening in *C. albicans* [65]. These data suggest that the *MED15* inactivation in *C. lusitaniae* induces cell wall stress, and that cell wall strengthening and reduced cell separation seem to be a conserved response to cell wall stress in *C. albicans* and *C. lusitaniae*. Up-regulation of 1,3-beta-glucanosyltransferase Pga4 and Glucan 1,3-beta-glucosidase Xog1 in the *med15*Δ mutant are in line with cell wall remodeling. Overall, our data suggest that the inactivation of *MED15* in *C. lusitaniae* results in proteomic changes that reflect adaptation to general stress, as well as pleiotropic phenotypes, such as defective filamentation, cell separation and mating.

### 4.2. The dpp3Δ Mutation Drove the Selection for the Additional Mutation in MED15

In two independent knockout strains, the mutation in *DPP3* was associated with an additional mutation in *MED15*. The mutation in *MED15* was more likely selected during the inactivation of *DPP3* rather than an artifact that would be attributable to the recipient strain or to the method of inactivation by transformation. First, the absence of *med15*Δ mutation was checked in the recipient strain by genome sequencing, suggesting that the mutation in *MED15* was selected when *DPP3* was inactivated. Second, the mutation in *MED15* occurred in two independent mutants generated using different strategies of inactivation (gene replacement and gene disruption). Selection of yeast transformants after the integration of the *URA3* marker in *DPP3* was performed on YNB supplemented with 1 M sorbitol. The mutation of *DPP3* might not be viable, for some unknown reason, without compensation by the *med15*Δ mutation on the selective medium. Other arguments support the idea that the inactivation of *DPP3* might be lethal or highly stressful to yeast during the selection of the *dpp3*Δ mutant if not compensated by the secondary mutation. Only a small number of transformants were obtained when attempting to delete the *DPP3* gene in the different recipient strains compared with the large number of transformants we usually obtain for other knockouts [25]. Furthermore, among them, few showed the correct integration of the inactivation cassette at the *DPP3* locus: 2 out of 43 transformants in the *ura3*Δ, *MAT***a** strain using the gene replacement strategy, and 1 out of 24 in the *ura3*Δ, *MAT***a** strain using the gene disruption strategy. On average, the homologous recombination rate for a gene inactivation in *C. lusitaniae* is 30% to 70%. On the other hand, the strain *dpp3*Δ *med15*Δ + *MED15* could be obtained using 5-FOA counterselection on YNB medium supplemented with URA, indicating the possibility of dissociating the *dpp3*Δ mutation from the *med15*Δ mutation, at least under certain conditions.

The selection of compensatory mutations following gene deletion has been previously described in *S. cerevisiae*, where every time *FIS1* was inactivated, a compensatory mutation was acquired [66]. Furthermore, a remarkable recent study used a collection of knockout strains in *S. cerevisiae* and showed that most of these strains presented mutations elsewhere in the genome [67]. The study pointed that a single-gene mutation may contribute to genomic imbalance, leading to adaptive mutations. Another study in which a collection of 143 mutants of *C. albicans* were analyzed indicated that as many as 10% of gene knockouts had an additional mutation that occurred during gene disruption, which was responsible for phenotypes not associated with the initially inactivated gene [68].

**Figure 8:**
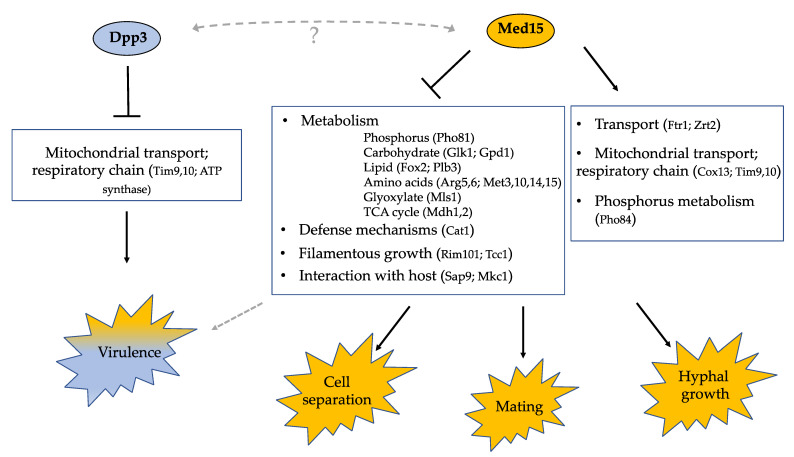
Summary of the functions of Med15 and Dpp3 in *C. lusitaniae* and of the biological pathways involved. Med15 is a subunit of the tail module of the mediator complex. Based on sequence homology with its orthologs in *S. cerevisiae* and *C. albicans*, Dpp3 is predicted to be a putative phosphatase in *C. lusitaniae*. The inactivation of *DPP3* repeatedly drove an additional mutation in *MED15.* Dpp3 was shown to play an important role in virulence [25]. In this study, we show that Med15 controls diverse cellular processes, such as cell separation, mating and hyphal growth. The role of Med15 in virulence was inferred from the partial restoration of virulence in the reconstituted strain *dpp3*Δ + *DPP3* but was not formally demonstrated. Proteomic analyses allowed to uncover the biological processes under the positive or negative regulation of Med15 and Dpp3. Dotted lines and question mark indicate that the links remain to be demonstrated.

How *DPP3* and *MED15* are connected remains to be discovered. Based on the functional categories that were enriched in the context of the *dpp3*Δ or the *med15*Δ mutation, we propose a model in which Dpp3 negatively controls a limited set of targets associated with mitochondrial transport and the respiratory chain, contributing greatly to virulence (Figure 8). *DPP3* was predicted to encode a phosphatase, so the impact on the expression of downstream targets is likely indirect. Notably, we found that the key transcriptional regulator of oxidative stress response Cap1 was down-regulated in the context of the *dpp3*Δ mutation. A mutant lacking *CAP1* in *C. albicans* was previously shown to have reduced viability when exposed to neutrophils or whole blood [69]. One hypothesis is that the mutation in the *DPP3* gene causes an imbalance in the mitochondria, strongly impacting virulence. Our data also suggest that Med15 controls a wide range of cellular processes that affect cell separation, mating and hyphal growth. Med15 is a negative regulator of specific genes involved in metabolism, defense mechanisms, filamentous growth and interaction with the host. Consistent with the pleiotropic effect of *med15*Δ mutation, key regulators of filamentation, cell polarity, stress and starvation response, such as the Rim101 and Tcc1 transcription factors, as well as the Mkc1 kinase, were found deregulated. Med15 also positively controls phosphorus metabolism and transport, as well as the mitochondrial respiratory chain. Thus, Med15 targets genes with a function in the mitochondria. It is interesting to note that *TIM9* and *TIM10* are common targets of Dpp3 and Med15, which regulate them in opposite ways (Figure 8). One hypothesis that can be proposed is that the mutation in *MED15* was selected to compensate for a detrimental imbalance in the mitochondria. The mutation in the tail subunit Med15 of the mediator likely directly impacts the recruitment of transcription factors and RNA Pol II, and would thus result in the loss of regulation of a large set of downstream targets and pleiotropic phenotypes.

### 4.3. Med15, a Hub That Integrates Metabolic and/or Mitochondrial Imbalances?

Although we have shown the importance of *DPP3* in the virulence of *C. lusitaniae*, the elucidation of other functions of *DPP3* in vitro was hampered by the systematic appearance of an additional mutation. In this paper, we achieved the goals we had set up for the current study. First, we have further characterized the phenotypes of the *dpp3*Δ mutant in terms of cell morphology and mating. Second, and more importantly, we identified the additional mutation in *MED15* that is responsible for the phenotypes, reporting for the first time a role for the mediator complex in cell separation, pseudohyphae formation and mating in *C. lusitaniae*.

Interestingly, this additional mutation did not occur only in the *dpp3*Δ mutant. A *ole2*Δ mutant, inactivated for a Δ9 fatty-acid desaturase that converts stearic acid to oleic acid, exhibited phenotypes comparable to those of the *dpp3*Δ, namely a defect in cell separation, pseudohyphae formation and mating (unpublished data). The *ole2*Δ + *OLE2* strain, reconstructed for an intact copy of *OLE2*, did not revert to wild-type phenotypes. When we sequenced the genome of the *ole2*Δ mutant, we found an additional mutation in the *MED15* gene. Furthermore, the reintroduction of a wild-type copy of *MED15* in the *ole2*Δ mutant rescued wild-type cell morphology and mating (unpublished data). This suggests that mutations occurring in genes related to lipid metabolism in *C. lusitaniae* may also result in an imbalance in cell metabolism, that would select for the additional *med15*Δ mutation. A link to mitochondria in the *ole2*Δ mutant is not established and requires further investigation, as does the interplay between a disturbance in lipid metabolism and Med15. That said, the mitochondrion being a dynamic compartment that interacts with diverse cellular organelles, including lipid droplets, and that integrates various stress signals that could be deregulated in both *dpp3*Δ and *ole2*Δ mutants. This mitochondrial dysfunction would only be viable for the cell if the Med15 subunit of the mediator complex is inactivated. Furthermore, the impact of metabolic alterations on the functions of the mitochondria was recently documented [70]. Understanding the underlying molecular mechanisms behind the selection of *med15*Δ mutation in such different mutants will be of primary importance in that it will provide a better understanding of mediator functions in *C. lusitaniae*.

## Figures and Tables

**Table 1:** *C. lusitaniae* strains used in this study.

Strain	Genotype	Description	Reference
WT	*MAT* **a**	Wild-type strain (6936)	ATCC38533
5094	*MAT*α	Wild-type strain	CBS collection, The Netherlands
CL38	*MAT*α	Clinical strain	[23]
*ura3*Δ	*ura3*Δ, *MAT***a**	Derived from 6936	[24]
*ura3*Δ *leu2*Δ	*ura3*Δ, *leu2*Δ, *MAT***a**	Derived from 6936	[24]
*leu2*Δ	*leu2*Δ, *MAT*α	Descendant from a cross between 5094 and *ura3*Δ *leu2*Δ	This study
*dpp3*Δ *ura3*Δ	*ura3*Δ, *dpp3*Δ::0, *med15*^mut^, *MAT***a**	*dpp3* knockout	[25]
*dpp3*Δ	*ura3*Δ::*URA3*, *dpp3*Δ::0, *med15*^mut^*MAT***a**	*dpp3* knockout	[25]
*dpp3*Δ + *DPP3* “REC”	*ura3*Δ::*URA3*, *dpp3*Δ::*DPP3*, *med15*^mut^*MAT***a**	*dpp3*Δ reconstituted for *DPP3*	[25]
*dpp3*Δ *med15*Δ	*ura3*Δ, *dpp3*Δ::0, *med15*Δ::*URA3, MAT***a**	*med15* knockout in the *dpp3*Δ mutant	This study
*dpp3*Δ *med15*Δ + *MED15*	*ura3*Δ, *dpp3*Δ::0, *med15*Δ::*MED15,* *MAT***a**	*dpp3*Δ *med15*Δ reconstituted for *MED15*	This study
*med15*Δ	*ura3*Δ, *med15*Δ::*URA3*, *MAT***a**	*med15* knockout	This study
*med15*Δ + *MED15*	*ura3*Δ, *med15*Δ::*MED15, MAT***a**	*med15*Δ reconstituted for *MED15*	This study
*dpp3*Δ^GD^*ura3*Δ	*ura3*Δ, *dpp3*Δ::*pGURA3-DPP3^130^,* *med15*^mut^, *MAT***a**	*dpp3* knockout	[25]

**Table 2:** Segregation analysis shows that *dpp3*Δ mutation is not responsible for the defective phenotypes in the *dpp3*Δ mutant. KW1-27: progeny of the cross between the *dpp3*Δ *ura3*Δ mutant (*ura3*Δ, *dpp3*Δ::0, *med15^mut^, MAT***a**) and the *leu2*Δ strain (*leu2*Δ, *MAT*α). REC (*ura3*Δ::*URA3, dpp3*Δ::*DPP3, med15^mut^, MAT***a**) is a reconstituted strain carrying a WT copy of the *DPP3* gene. For each strain, the genotype at the *DPP3* locus was determined by PCR. The cytokinesis phenotype was observed using the microscope after 24 h in a 5 mL YPD liquid culture at 35 °C, 215 rpm. The pseudohyphal growth phenotype was assessed by spotting 5 μL of an overnight YPD culture of each strain (1 × 10^7^ cells standard inoculum) on YCB agar plates. After 24 h of incubation at 30 °C, microscopic images were taken and pseudohyphae length was measured. The mating phenotype was assessed by crossing each strain with reference strains 6936 (*MAT***a**) and 5094 (*MAT*α) on YCB agar plates. After 72 h, mating cells were observed between slides and coverslips for typical mating structures. Progeny were assigned the “WT” or “KO” phenotype in reference to the phenotypes of the parental strains.

Strain	Genotype at *DPP3* Locus	Cell Separation	Pseudohyphal Growth	Mating
WT	*DPP3*	WT	WT	WT
*dpp3*Δ	*dpp3*Δ	KO	KO	KO
REC	*DPP3*	KO	KO	KO
KW1	*dpp3*Δ	WT	WT	WT
KW2	*dpp3*Δ	WT	WT	WT
KW3	*DPP3/dpp3*Δ	WT	WT	WT
KW4	*DPP3*	KO	KO	KO
KW5	*DPP3*	KO	KO	KO
KW6	*DPP3/dpp3*Δ	WT	WT	WT
KW7	*DPP3*	KO	KO	KO
KW8	*DPP3*	WT	WT	WT
KW9	*DPP3*	WT	WT	WT
KW10	*DPP3*	KO	KO	KO
KW11	*DPP3*	WT	WT	WT
KW12	*DPP3*	WT	WT	WT
KW13	*dpp3*Δ	KO	KO	KO
KW14	*DPP3*	WT	WT	WT
KW15	*DPP3*	WT	WT	WT
KW16	*DPP3*	KO	KO	KO
KW17	*dpp3*Δ	WT	WT	WT
KW18	*DPP3*	WT	WT	WT
KW19	*DPP3*	WT	WT	WT
KW20	*dpp3*Δ	WT	WT	WT
KW21	*dpp3*Δ	WT	WT	WT
KW22	*DPP3*	WT	WT	WT
KW23	*DPP3/dpp3*Δ	WT	WT	WT
KW24	*DPP3/dpp3*Δ	WT	WT	WT
KW25	*dpp3*Δ	WT	WT	WT
KW26	*DPP3/dpp3*Δ	WT	WT	WT
KW27	*dpp3*Δ	WT	WT	WT

**Table 3:** Enrichment analysis of the proteins up-regulated in the REC strain (carrying the *med15*Δ mutation) compared with the WT strain. We used DAVID tool (https://david.ncifcrf.gov, accessed on 21 February 2023) with the GO term biological process (A) or KEGG pathway (B) category and *C. lusitaniae* ATCC42720 as the reference proteome. *p* value (Ease score) cut-off was set up at 0.05. The table shows only minimally overlapping GO terms.

**(A)**				
**Term**	**Count**	**%**	***p* Value**	**Fold Enrichment**
GO:0005975~carbohydrate metabolic process	10	8.5	8.6 × 10^−4^	3.8
GO:0006099~tricarboxylic acid cycle	4	3.4	6.1 × 10^−3^	10.2
GO:0044242~cellular lipid catabolic process	4	3.4	7.9 × 10^−3^	9.3
GO:0006979~response to oxidative stress	3	2.6	7.9 × 10^−3^	20.7
GO:0000096~sulfur amino acid metabolic process	4	3.4	1.0 × 10^−2^	8.5
GO:0009060~aerobic respiration	4	3.4	1.4 × 10^−2^	7.5
GO:0015980~energy derivation by oxidation of organic compounds	5	4.3	2.6 × 10^−2^	4.3
GO:0046434~organophosphate catabolic process	3	2.6	2.8 × 10^−2^	11.0
**(B)**				
**Term**	**Count**	**%**	***p* Value**	**Fold Enrichment**
clu01100:Metabolic pathways	44	37.6	2.0 × 10^−7^	1.8
clu00630:Glyoxylate and dicarboxylate metabolism	8	6.8	7.7 × 10^−6^	9.8
clu01200:Carbon metabolism	12	10.3	1.4 × 10^−4^	3.9
clu00620:Pyruvate metabolism	9	7.7	1.8 × 10^−4^	5.3
clu04146:Peroxisome	8	6.8	5.0 × 10^−4^	5.3
clu01110:Biosynthesis of secondary metabolites	21	17.9	1.1 × 10^−3^	2.0
clu00040:Pentose and glucuronate interconversions	4	3.4	4.4 × 10^−3^	11.1
clu00640:Propanoate metabolism	4	3.4	3.6 × 10^−2^	5.3

## Data Availability

The data presented in this study are included in the main text and Supplementary Materials. Mass spectrometry proteomics data were deposited to the ProteomeXchange Consortium (http://proteomecentral.proteomexchange.org accessed on 3 July 2020) via the PRIDE partner repository [31] with the dataset identifier PXD020220.

## Supplementary Materials

The following supporting information can be downloaded at: https://www.mdpi.com/article/10.3390/jof9030333/s1, Figure S1: cell morphology of the WT and *dpp3*Δ strains in liquid culture; Figure S2: pseudohyphal growth of the WT and *dpp3*Δ strains; Figure S3: effect of farnesol on *C. lusitaniae* and *C. albicans* hyphal growth on their respective inducing media YCB and RPMI; Figure S4. alignments of the *MED15* gene and the corresponding Med15 protein in the WT strain and the *dpp3*∆ mutant. Table S1: primers used in this study; Table S2: analysis of the variants specific to the *ura3*∆ recipient strain when compared with the WT reference strain 6936 (Table S2.1), and specific to the *dpp3*Δ mutant when compared with the *ura3*∆ recipient strain (Table S2.2); Table S3: list of proteins distinctly abundant in the *dpp3*Δ mutant (KO) carrying the *dpp3*Δ and *med15*Δ mutations compared with the reconstituted strain (REC) carrying the *med15*Δ mutation only; Table S4: list of distinctly abundant proteins in the reconstituted strain (REC) harboring the *med15*Δ mutation compared with the wild-type (WT) strain; Table S5: summary of all reported phenotypes for the *dpp3*Δ mutant and responsible mutations.
