# Supplementary material for: Mediator Subunit Med15 Regulates Cell Morphology and Mating in Candida lusitaniae
Source: J Fungi (Basel). 2023 Mar 8;9(3):333. doi: 10.3390/jof9030333 (PMC10053558; doi:10.3390/jof9030333)
Supplement: Supplementary file 1 [file jof-09-00333-s001.zip › Suppl Figure Legends.pdf]

Supplementary figure legends

Figure S1. Cell morphology of the WT and *dpp3*Δ strains in liquid culture.

Cells from the WT and the *dpp3*Δ strains were grown in 5 ml YPD liquid media at 35°C, 215 rpm and observed by microscopy using DIC for bright field at 3 h, 6 h, and 24 h (**A**) or the DAPI filter to image the Calcofluor fluorescence at 24 h (**B**). CFW showed increased fluorescence intensity at the bud necks and at the separation between mother and daughter cells. The *dpp3*Δ mutant forms multi-cell chains that are typical of a cell separation defect. Scale bars represent 10 µm.

Figure S2. Pseudohyphal growth of the WT and *dpp3*Δ strains.

Yeast aliquots from the wild-type and the *dpp3*Δ strains were spotted on agar plates containing the filamentation-inducing medium YCB. After 24 h of incubation at 30°C, images of the outlines of colonies were taken using microscopy. Inserts show the corresponding cells observed between slide and cover slip. Long and short scale bars represent 300 µm and 10 µm respectively.

Figure S3. Effect of farnesol on *C. lusitaniae* and *C. albicans* hyphal growth on their respective inducing media YCB and RPMI. Images were taken after 48 h of incubation at 30°C and show colony outlines. Scale bars represent 300 µm. Farnesol was added to the agar plates before spotting *C. lusitaniae* (a-f) or *C. albicans* (g-h) cells. Hyphal growth of *C. albicans* (WT strain SC5314) was inhibited by 200 µM of farnesol on RPMI, and was uesd as a positive control (h). Note that 200 µM of farnesol did not affect the growth of *C. lusitaniae* pseudohyphae on its inducing medium YCB (b). In contrast, pseudohyphal growth of *C. lusitaniae* was induced by 200 µM of farnesol on the otherwise non-inducing medium RPMI (d), whereas no effect was observed on the *dpp3*Δ mutant (f).

Figure S4. Sequence alignment of the *MED15* gene and the corresponding Med15 protein in the WT strain and the *dpp3*Δ mutant. (**A**) nucleotide alignment showing only the surrounding region of the 19 bp insertion in the mutated *MED15* allele; (**B**) alignment of the whole amino

acid sequences showing the frameshift insertion in SER365 that results in a premature stop codon at position 376 and a truncated Med15 protein in the *dpp3*Δ mutant. “KIX” domain (activator-binding domain, pfam 16987), “GAL11 coactivator” domain (cd12191), “MED15” domain (mediator complex subunit 15, pfam05397), “PHA03378” domain (Epstein-Barr virus nuclear antigen 3 (EBNA-3), cl27975).
