# Supplementary material for: Mediator Subunit Med15 Regulates Cell Morphology and Mating in Candida lusitaniae
Source: J Fungi (Basel). 2023 Mar 8;9(3):333. doi: 10.3390/jof9030333 (PMC10053558; doi:10.3390/jof9030333)
Supplement: Supplementary file 1 [file jof-09-00333-s001.zip › Table S1_primers.pdf]

Table S1. Primers used in this study.

Primer	Sequence 5' – 3'	Use
5AmDpp3_800	AGTACGGGATCTCATCTGTTC	Genotyping of the *DPP3* locus
3AvDpp3_521	GGCTATCATGTCTGAAAGCG	Genotyping of the *DPP3* locus
B584	CATGGGCAATATTTCCAACG	Genotyping of the *MED15* locus
B586	GCCATATTGTTGGAGTTATTAAC	Genotyping of the *MED15* locus
B343	AACAACAGCACCAACAACAC	Amplification of the final *MED15* inactivation cassette
B344	AGAAGATCAGGTCCAGCAAG	Amplification of the final *MED15* inactivation cassette
B413	CGGTACCCGGGGATCAACAACAGCACCAACAACAC	Amplification of *MED15* upstream region for the inactivation cassette
B414	TCTGCCATCAACGGGCCTCCTAGTTGACCAACACC	Amplification of *MED15* upstream region for the inactivation cassette
B415	GAGAATGTCATCTCGAGCTAACGGTTCCATTTCAC	Amplification of *MED15* downstream region for the inactivation cassette
B416	CGACTCTAGAGGATCAGAAGATCAGGTCCAGCAAG	Amplification of *MED15* downstream region for the inactivation cassette
B419	TACTATGGCGCAAGAGTTTG	Amplification of a wild-type copy of *MED15*
B420	CATCTGATTCATCCGTTGAG	Amplification of a wild-type copy of *MED15*
B280	CCCGTTGATGGCAGAGTTGG	Amplification of *URA3* fragment for the inactivation cassette
B238	CGAGATGACATTCTCCATTC	Amplification of *URA3* fragment for the inactivation cassette
