# Supplementary material for: Mediator Subunit Med15 Regulates Cell Morphology and Mating in Candida lusitaniae
Source: J Fungi (Basel). 2023 Mar 8;9(3):333. doi: 10.3390/jof9030333 (PMC10053558; doi:10.3390/jof9030333)
Supplement: Supplementary file 1 [file jof-09-00333-s001.zip › Table S2_SNP.pdf]

Table S2. Analysis of the variants specific to the *ura3*Δ recipient strain when compared to the WT reference strain 6936 (Table S2.1), and specific to the *dpp3*Δ mutant when compared to the *ura3*Δ recipient strain (Table S2.2). REF”: allele of reference strain 6936, “ALT”: alternative allele, Variant type and predictive impact as annotated in SnpEff (https://pcingola.github.io/SnpEff/), “CDS POS”: polymorphism position from the ATG codon, AA modification: “fs” for frameshift, “del” for deletion.

Table S2.1

#CHROM	POS	REF	ALT	Variant type	Predictive impact	Locus	CDS POS	AA modification	Region	Description
LYUB02000005	462376	T	G	missense	Moderate	A9F13_05g02464	2858	Lys953Thr	ORF	uncharacterized protein
LYUB02000006	102540	A	G	upstream gene	Modifier	A9F13_06g00594	-176	none	5UTR	putative 37S ribosomal protein
LYUB02000007	105090	T	G	missense	Moderate	A9F13_07g00583	1156	Ser386Ala	ORF	putative branchpoint-bridging protein
LYUB02000012	226151	C	T	upstream gene	Modifier	A9F13_12g01265	-919	none	5UTR	uncharacterized protein
LYUB02000044	1493	A	C	intergenic region	Modifier	none	/	/	intergenic	
LYUB02000044	1488	T	TGCACGCCTTCTAAGATAACGGGCTCCGGTTTTAAAGCCGTTGGTTATAG	intergenic region	Modifier	none	/	/	intergenic	

Table S2.2

#CHROM	POS	REF	ALT	Variant type	Predictive impact	Locus	CDS POS	AA modification	Region	Description
LYUB02000020	140610	G	GAGGAATGAACAACATGAG	frameshift insertion	High	A9F13_20g00990	1076	Ser365fs	ORF	putative Gal11
LYUB02000011	341777	CTA	C	upstream gene	Modifier	A9F13_11g01958	-941_-940	none	5UTR	putative Alr1
LYUB02000013	103535	GAA	G	upstream gene	Modifier	A9F13_13g00649	-136_-135	none	5UTR	putative Avt1
LYUB02000039	1023	TTTGAACTCGAGGTTTCGC	T	intron	Modifier	A9F13_41g00066	1241_1224	none	intron	putative Hyr3
LYUB02000005	517344	AATCGAGAGAATCGAGAGAATCGAGAG	G	disruptive inframe deletion	Moderate	A9F13_05g02640	125_151	Glu42_Arg50del	ORF	uncharacterized protein
LYUB02000026	106364	GGACAAGTCTGACAAGTCTGACAAGTCT	G	disruptive inframe deletion	Moderate	A9F13_26g00638	158_184	Asp53_Ser61del	ORF	uncharacterized protein
