# Supplementary material for: Mediator Subunit Med15 Regulates Cell Morphology and Mating in Candida lusitaniae
Source: J Fungi (Basel). 2023 Mar 8;9(3):333. doi: 10.3390/jof9030333 (PMC10053558; doi:10.3390/jof9030333)
Supplement: Supplementary file 1 [file jof-09-00333-s001.zip › Table S5_Summary.pdf]

Table S5: Summary of all reported phenotypes for the *dpp3*Δ mutant and responsible mutations. For the defective phenotypes examined in our previous study, attribution to loss of Med15 was inferred from the lack of restoration of WT phenotypes in the *dpp3*Δ mutant after reconstruction of a *DPP3* WT locus, but was not formally demonstrated.

***dpp3*Δ phenotype**	**Due to the loss of**	**Study**
Intracellular pyrophosphate decreased	Med15	Sabra *et al.* 2014
PEA secretion decreased	Med15	Sabra *et al.* 2014
Tyrosol secretion increased	Med15	Sabra *et al.* 2014
Yeast survival in macrophages increased	Med15	Sabra *et al.* 2014
ROS production by macrophages decreased	Med15	Sabra *et al.* 2014
TNF-α secretion by macrophages increased	Med15	Sabra *et al.* 2014
IL-10 secretion by macrophages decreased	Med15	Sabra *et al.* 2014
Virulence in mice decreased	Dpp3 and Med15	Sabra *et al.* 2014
TNF-α secretion in mice decreased	Dpp3 and Med15	Sabra *et al.* 2014
Colonization of mouse brains decreased	Dpp3 and Med15	Sabra *et al.* 2014
Cell separation decreased	Med15	Current work
Hyphal growth decreased	Med15	Current work
Mating decreased	Med15	Current work
