# Supplementary figures and images for: Mediator Subunit Med15 Regulates Cell Morphology and Mating in Candida lusitaniae
Source: J Fungi (Basel). 2023 Mar 8;9(3):333. doi: 10.3390/jof9030333 (PMC10053558; doi:10.3390/jof9030333)

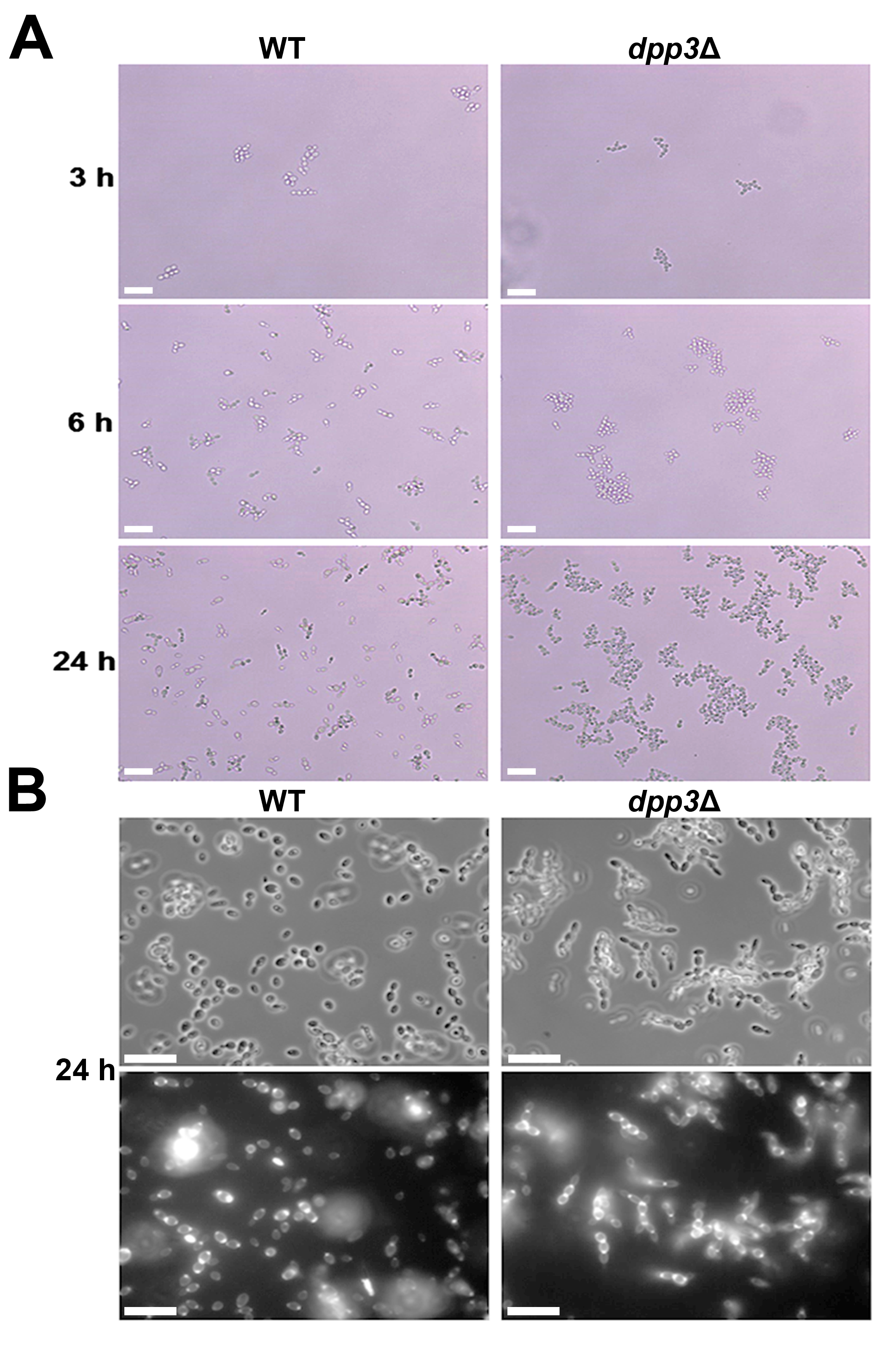

Supplement: Supplementary file 1 [file jof-09-00333-s001.zip › Figure S1_Clusters.tif]

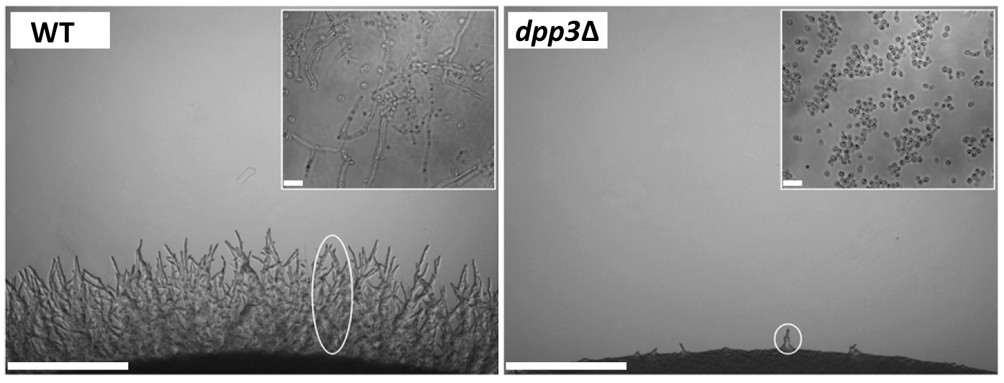

Supplement: Supplementary file 1 [file jof-09-00333-s001.zip › Figure S2_hyphalgrowth.tif]

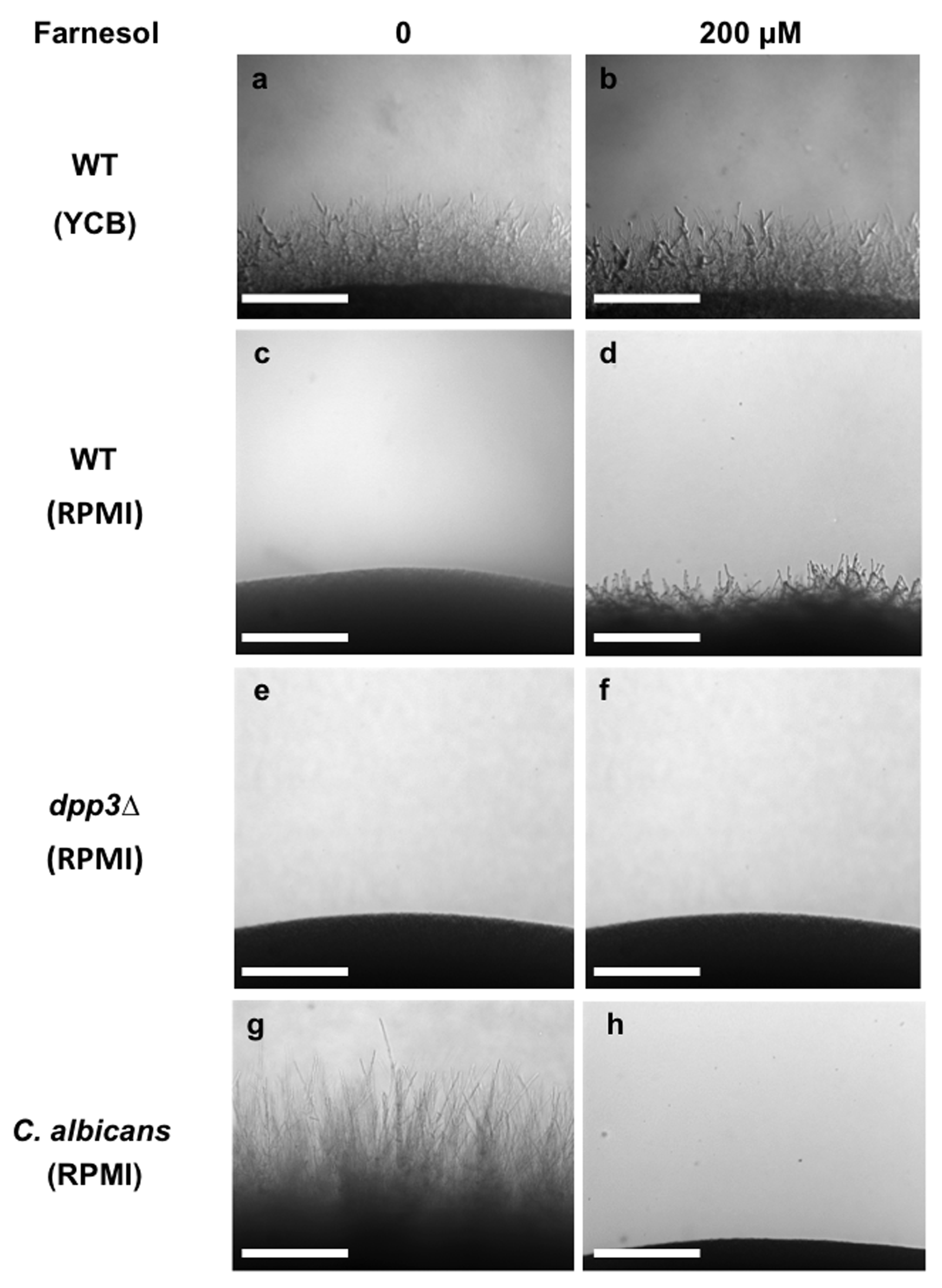

Supplement: Supplementary file 1 [file jof-09-00333-s001.zip › Figure S3.tif]

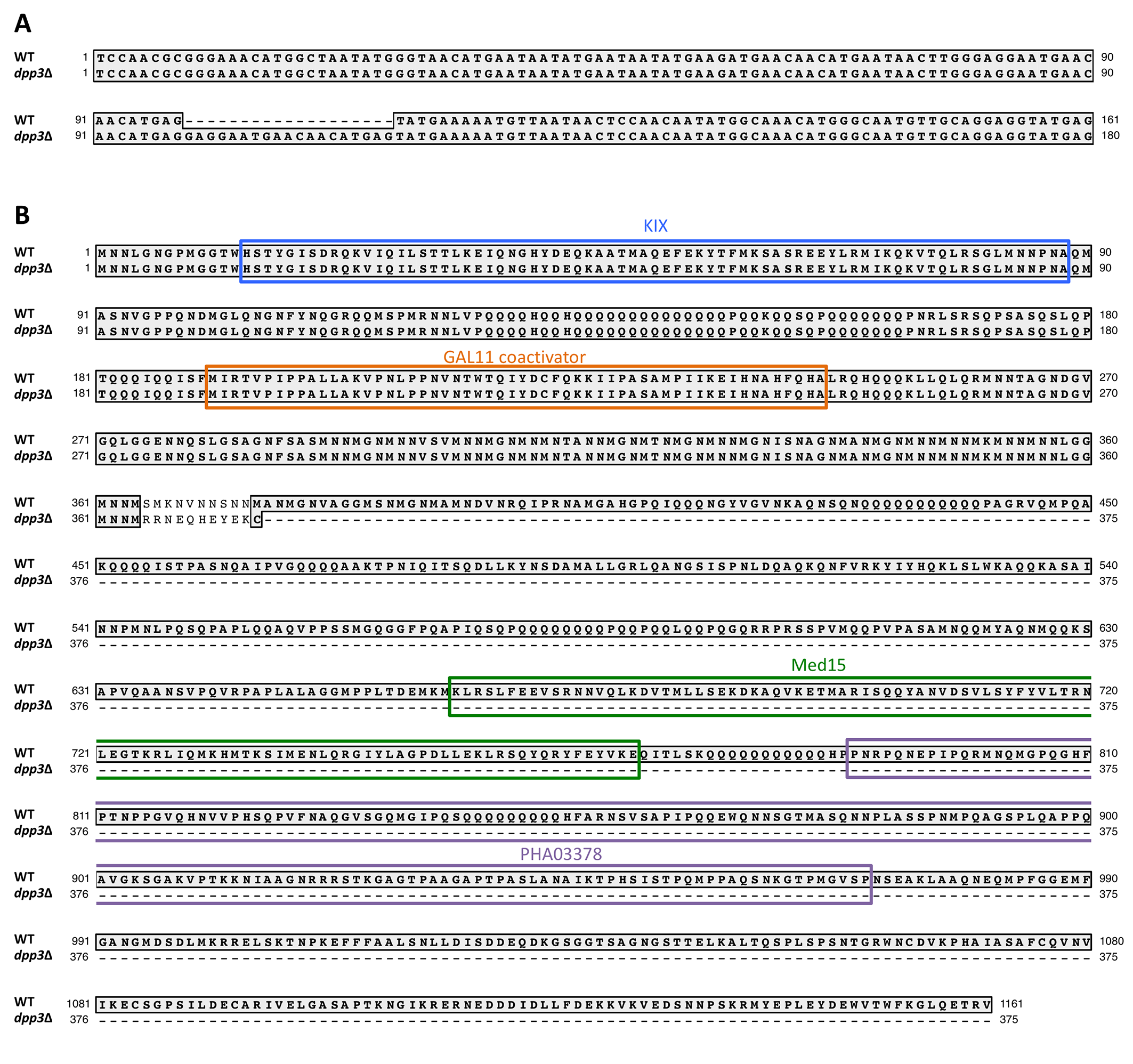

Supplement: Supplementary file 1 [file jof-09-00333-s001.zip › Figure S4_Alignments.tif]
